# Comparison of Treatment Approaches and Subsequent Outcomes within a Pulmonary Embolism Response Team Registry

**DOI:** 10.1155/2024/5590805

**Published:** 2024-03-22

**Authors:** Anthony J. Weekes, Ariana Trautmann, Parker L. Hambright, Shane Ali, Angela M. Pikus, Nicole Wellinsky, Kelly L. Goonan, Sarah Bradford, Nathaniel S. O'Connell

**Affiliations:** ^1^Department of Emergency Medicine, Atrium Health's Carolinas Medical Center, Charlotte, North Carolina, USA; ^2^Tulane University School of Medicine, New Orleans, Louisiana, USA; ^3^Department of Biostatistics and Data Science, Wake Forest School of Medicine, Winston-Salem, North Carolina, USA

## Abstract

**Objectives:**

To characterize the association between pulmonary embolism (PE) severity and bleeding risk with treatment approaches, outcomes, and complications.

**Methods:**

Secondary analysis of an 11-hospital registry of adult ED patients treated by a PE response team (August 2016–November 2022). Predictors were PE severity and bleeding risk. The primary outcome was treatment approach: anticoagulation monotherapy vs. advanced intervention (categorized as “immediate” or “delayed” based on whether the intervention was received within 12 hours of PE diagnosis or not). Secondary outcomes were death, clinical deterioration, and major bleeding.

**Results:**

Of the 1832 patients, 139 (7.6%), 977 (53.3%), and 9 (0.5%) were classified as high-risk, intermediate-high, intermediate-low, and low-risk severity, respectively. There were 94 deaths (5.1%) and 218 patients (11.9%) had one or more clinical deterioration events. Advanced interventions were administered to 86 (61.9%), 195 (27.6%), and 109 (11.2%) patients with high-risk, intermediate-high, and intermediate-low severity, respectively.Major bleeding occurred in 61/1440 (4.2%) on ACm versus 169/392 (7.6%) with advanced interventions (p <0.001): bleeding withcatheter-directed thrombolysiswas 19/145 (13.1%) versus 33/154(21.4%) with systemic thrombolysis,p= 0.07. High risk was twice as strong as intermediate-high risk for association with advanced intervention (OR: 5.3 (4.2 and 6.9) vs. 1.9 (1.6 and 2.2)). High risk (OR: 56.3 (32.0 and 99.2) and intermediate-high risk (OR: 2.6 (1.7 and 4.0)) were strong predictors of clinical deterioration. Major bleeding was significantly associated with advanced interventions (OR: 5.2 (3.5 and 7.8) for immediate, 3.3 (1.8 and 6.2)) for delayed, and high-risk PE severity (OR: 3.4 (1.9 and 5.8)).

**Conclusions:**

Advanced intervention use was associated with high-acuity patients experiencing death, clinical deterioration, and major bleeding with a trend towards less bleeding with catheter-directed interventions versus systemic thrombolysis.

## 1. Introduction

Evidence-based treatment recommendations for confirmed pulmonary embolism (PE) are based on a patient's clinical presentation and risk classification for in-hospital or 30-day death [1, 2]. Contemporary treatment approaches include (1) anticoagulation monotherapy, (2) anticoagulation with close monitoring for the need for subsequent advanced intervention (“watch and wait”), and (3) immediate use of advanced interventions. For high-risk PE patients without high bleeding risk, the guidelines recommend systemic thrombolysis with anticoagulation. It is debatable whether the benefits of systemic thrombolysis outweigh the risks for those without high-risk PE features. Management of intermediate-risk PE patients is less straightforward. For intermediate-risk PE patients, expert opinion recommends against an advanced intervention like systemic thrombolysis when given a binary choice of systemic thrombolysis with plasminogen activators versus anticoagulation monotherapy [1, 3–5]. However, the European Society of Cardiology (ESC) recommends advanced interventions beyond just systemic thrombolysis for patients who suffer clinical deterioration despite anticoagulation monotherapy [1]. In practice, physicians administer anticoagulation and hold off giving systemic thrombolysis until intermediate-risk PE patients subsequently deteriorate.

Other advanced PE interventions, such as catheter-directed treatment (CDT), provide focused antithrombotic treatment either by targeted delivery of thrombolytic medications, disruption or extraction of the offending thrombi, or a combination of approaches (pharmacomechanical). Evidence is emerging that major bleeding complications decrease and mortality benefits increase when these other advanced PE interventions are used [6, 7]. In our experience, advanced PE interventions are not predictably given to patients with high-risk PE. Furthermore, eligibility for advanced PE interventions has broadened to include high-risk PE with high bleeding risk and intermediate-risk PE with features of distress and/or transitioning to high-risk PE.

Despite increased options for advanced PE interventions and the presence of multidisciplinary PE response teams (PERT), there is considerable variation in practice [8]. Even within the same healthcare system, differences in physician decision-making and hospital-specific availability of advanced PE interventions may result in different care provided by different physician teams with different clinical and safety outcomes for patients. The primary objective of this study was to characterize the association between PE severity and bleeding risk with treatment approach (anticoagulation monotherapy versus delayed advanced intervention versus immediate advanced intervention). Our secondary objectives were to compare outcomes and complications between treatment approaches and determine how treatment approaches and morbidity outcomes themselves are associated with death.

## 2. Methods

### 2.1. Study Design and Setting

We studied patient characteristics and outcomes from the Clinical Outcomes in Pulmonary Embolism Research Registry (COPERR), which was approved by the Atrium Health Institutional Review Board. COPERR is an observational registry of adult patients treated by a multidisciplinary PERT in 11 emergency departments (EDs) within the Atrium Health system in North Carolina, USA [9]. PE care delivery at Atrium Health (inpatient and ED) is supported by an established PE management algorithm and PERT. Each participating ED has systemic thrombolysis available as an advanced PE intervention for those at high-risk/massive PE. However, only three of the 11 hospitals are fully equipped to provide further advanced PE interventions, with multidisciplinary support (vascular surgery, cardiothoracic surgery, and interventional cardiology) and intensive care units (ICUs). These sites were considered PE referral hospitals. The remaining EDs transfer intermediate-high and high-risk PE patients to one of the three PE referral sites. The PERT program holds regular meetings with medical directors and clinical experts, which cover quality assurance and review of clinical care metrics specific to PE clinical management and outcomes. PERT notification leads to triaging by a designated clinician to expand or narrow the number of multidisciplinary team members notified.

### 2.2. Study Population

Our study population was patients with acute PE entered into the registry between August 2016 and November 2022. Inclusion criteria were adult ED patients (≥age 18) with confirmed PE, who met intermediate- or high-risk PE criteria or for whom the PERT was activated [1, 2, 10].

### 2.3. Study Protocol

We collected data on demographics, vital signs, comorbidities, laboratory values, and imaging features, including bedside and comprehensive echocardiography studies and computed tomography (CT). We also captured performance metrics for the 11 participating hospitals, including overall mean time from PE diagnosis to first heparin and mean time from PERT activation to first heparin, as well as mean hospital length of stay.

#### 2.3.1. PE Severity Classification

We used PE severity assignments as defined by the American College of Chest Physicians (ACCP), American Heart Association (AHA), and European Society of Cardiology (ESC) [1, 2, 10]. AHA classifications of massive, severe submassive, and nonsevere submassive correspond to ESC classifications of high-risk, intermediate-high-risk, and intermediate-low-risk PE, respectively. Throughout this manuscript, we use the ESC nomenclature.

We modified ESC and AHA definitions to require confirmation of the presence of right ventricular (RV) dilatation in the absence of primary unstable dysrhythmia or other causes, such as severe sepsis (although conditions may coexist).  Table S1 shows the definitions and classification criteria for PE severity.

#### 2.3.2. Bleeding Risk Assessment

We adapted known bleeding risk tools [2, 11–13].  Table S1 shows the criteria used for bleeding risk assessment.

#### 2.3.3. Eligibility for Advanced PE Intervention

To determine eligibility for one or more advanced PE interventions, we followed recommendations of the ACCP, ESC, and American Society of Hematology for high-risk and intermediate-risk PE patients [1, 3–5]. As a step further, we profiled patients into six categories according to the couplets of PE risk classification and bleeding risk assignments at the presentation. A patient was deemed eligible for an advanced PE intervention if there were no recommendations against the intervention in the patient's profile. As shown in Table S2, high-risk and intermediate-high-risk patients were deemed eligible for advanced PE intervention.

### 2.4. Key Outcome Measures

Our primary outcome was the treatment approach, expressed ordinally as anticoagulation monotherapy (“watch and wait”), delayed advanced PE intervention (meaning that the intervention was administered for more than 12 hours after PE diagnosis), and immediate advanced PE intervention (meaning that intervention was administered within 12 hours of PE diagnosis). We used the time of electronic order entry of medication or the documented start time of procedural interventions to signify the timing of advanced PE intervention. We chose the 12-hour cut-off based on a study that reported the impact of a treatment algorithm and PERT on PE interventions. In that study, improved quality measures were associated with the delivery of advanced PE interventions within a few hours compared with over 12 hours from PE diagnosis [14]. We used a 12-hour cutoff distinguish delayed versus immediate advanced interventions to factor in time for PERT discussions and initiation of more resource intensive interventions such as CDT which are not as immediate as systemic thrombolysis. This ordinal approach lends more granularity to decision-making and represents real-world decision-making and hesitation.

The types of advanced PE interventions were systemic thrombolysis with plasminogen activators, CDT, surgical embolectomy, and mechanical circulatory support with venoarterial extracorporeal membrane oxygenation (ECMO). Systemic thrombolysis included a full dose of alteplase 100 mg over 2 hours or tenecteplase 40 to 50 mg bolus, or a reduced dose of alteplase 50 mg over 2 hours. CDT included catheter-directed thrombolysis, catheter-based embolectomy (large bore and small bore), aspiration thrombectomy, and mechanical thrombectomy.

Our secondary outcomes were in-hospital clinical deterioration (including death) and major bleeding complications. The main and competing concerns clinicians have with managing patients with intermediate- or high-risk PE are acute clinical deterioration (if the patient is not treated with advanced PE interventions) and major bleeding (if the patient is treated with advanced PE interventions). We defined clinical deterioration as cardiac arrest, unscheduled rescue mechanical ventilation or positive pressure ventilation, administration of vasoactive medication for hypotension, ECMO, right ventricular assist device, or death. Persistence of hemodynamic instability (applicable to those designated as high-risk PE) after initial ED presentation to hospital admission was considered clinical deterioration. We reported PE-related deaths during the initial hospitalization. Death was PE-related if the treating physician's documentation determined the cause of death to be definitely or likely caused by PE.

We used the International Society on Thrombosis and Hemostasis definition of major bleeding, which is defined as symptomatic bleeding in a critical organ area, bleeding causing a fall in the hemoglobin level of greater than 2 g/dL, or fatal bleeding. Hypotension associated with major bleeding from PE intervention was classified as major bleeding rather than PE-related clinical deterioration. If major bleeding occurred, we determined if it was associated with anticoagulation monotherapy or advanced PE interventions, including thrombolysis.

### 2.5. Data Analysis

Descriptive statistics, including means and standard deviations or counts and percentages, were calculated. Missingness was reported. We reported univariate statistics stratified by primary (treatment approach) and secondary outcomes. To estimate differences statistically in each univariate case, we used ANOVA to compare treatment approaches with respect to continuous variables and chi-square tests for categorical variables. R and RStudio software were used for all analyses [15]. A two-tailed value of less than 0.05 was considered statistically significant in a univariate sense.

For multivariate analyses, we used a regression model for the ordinal outcome of the treatment approach (anticoagulation monotherapy versus delayed advanced PE intervention (>12 hours after PE diagnosis) versus immediate advanced PE intervention (≤12 hours of PE diagnosis)) by PE severity risk and bleeding risk assessment. Dickey et al. demonstrated that modeling of ordinal outcomes as opposed to binary outcomes when it comes to clinical variables can lead to increased statistical power [16].

We dichotomized the ordinal outcome and fit it via a logistic regression mixed effects model, controlling for hospital sites with random intercepts. We assessed an interaction effect between PE risk and bleeding risk assessment and used multivariate analyses to differentiate any interaction of these predictors of interest on the primary outcome. We first fit simple models for the relationship between treatment approach and clinical deterioration. Then, we fit adjusted models with PE severity risk and bleeding risk added in and controlled for hospital sites with random intercepts.

## 3. Results

### 3.1. Patient and Hospital Characteristics

**Figure 1:**
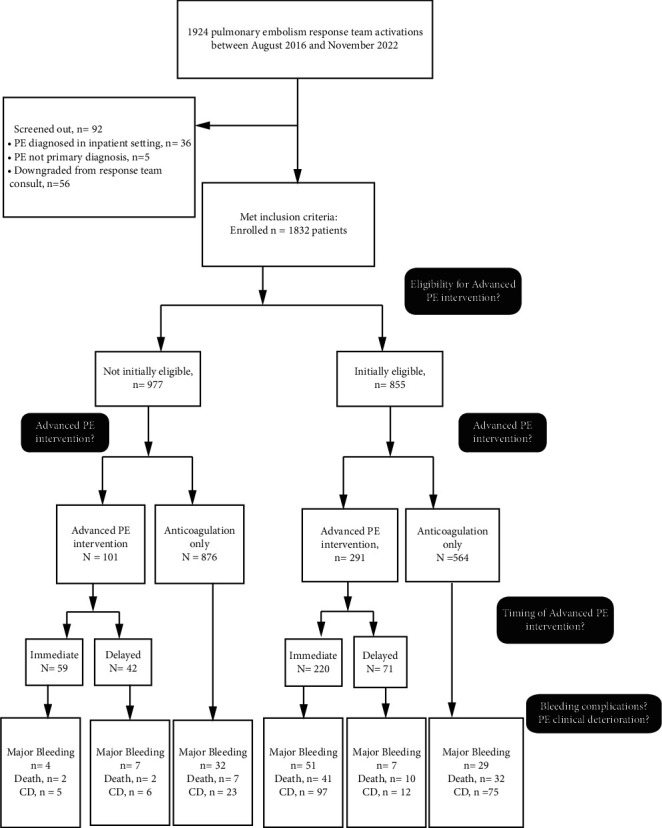
Study flow diagram.

Of the 1924 registry patients screened (August 2016–November 2022), 1832 PE patients met the criteria for complete analysis from 11 regional EDs (Figure 1). As shown in Table 1, the mean age of our study population was 62.8 (SD 16.0) years, 51.4% were female, 61.4% were Caucasian, and 35% were African-American. PE risk factors included prior PE or deep venous thrombosis diagnosis (23.3%), recent hospitalization (14.5%), any malignancy (11.5%), recent surgery (9.0%), family history of venous thromboembolism (9.0%), hormone replacement therapy (6.0%), limb immobilization (4.8%), clotting disorder (3.3%), and recent trauma (2.5%). PE severity classifications at presentation to ED were 7.6% high-risk, 38.6% intermediate-high risk, and 53.3% intermediate-low risk. Fifty-nine (42.4%) of the 139 high-risk PE patients had hemodynamic collapse/cardiac arrest at initial ED presentation. Most patients had RV dilatation by CT or echocardiography, more than one-half had elevated cardiac biomarkers, more than one-third had hypoxia with respiratory distress, and almost one in five had elevated shock index with a small percentage having hemodynamic instability. Bleeding risk was classified as high for 13.5%, moderate for 49.9%, and low for 36.5% of the 1832 patients.

Table S3 shows patient characteristics and treatments given at the different participating hospitals. Five hospitals accounted for 90% of patients in this report (hospitals A–E). Hospitals contributing less than 100 patients to the registry were grouped as “other hospitals.” There were significant differences in race and age of patients between hospitals. There were significant differences across hospitals A–E in proportions of patients with high bleeding risk, receiving ICU level of care, and hemodynamic collapse/cardiac arrest at presentation and in PE severity/bleeding risk assessment profiles. There was a notable difference in the use of advanced PE interventions between hospitals A and E. Likewise, there were significant variations in hospitals' use of systemic thrombolysis versus CDT. For example, we looked at two of the PE referral sites and noted for hospital A that 138 patients had one or more advanced PE interventions compared to 111 patients at hospital D. However, at hospital A, there were 8 CDTs compared to 68 at hospital D. The mean time from PE diagnosis to first heparin for all 11 hospitals was 115 minutes, and the mean time from PERT activation to first heparin was 83.5 minutes. The mean hospital length of stay was 5.66 (11.4) days.

### 3.2. Main Findings

As shown in Figure 1, 855 of 1832 patients (46.7%) were deemed eligible for one or more advanced PE interventions at presentation. Of the 855, 564 (66%) received anticoagulation monotherapy during hospitalization, while 291 (34%) received one or more advanced PE interventions. For 71 of the 291 (24%) patients, the advanced PE intervention was delayed for >12 hours from PE diagnosis. Of the 977 patients considered ineligible for advanced PE intervention, 101 (10%) eventually had one or more advanced PE interventions during hospitalization. For 42 of the 101 (42%) patients, the start of advanced PE intervention was delayed for >12 hours after PE diagnosis.

#### 3.2.1. Primary Outcome

Table 1 shows a univariate analysis of patient characteristics grouped *by treatment approach* and expressed as an ordinal outcome. Of the 392 patients who received an advanced PE intervention, 154 (39.3%) had systemic thrombolysis, 147 (37.5%) had CDT, 10 (2.6%) had ECMO, and 9 (2.3%) had surgical embolectomy. Some had more than one type of advanced PE intervention. Over 90% of CDTs were ultrasound-assisted catheter-directed thrombolysis versus aspiration/mechanical thrombectomy (6.0%), catheter-directed thrombolysis without ultrasound assistance (2.7%), and aspiration thrombectomy (2.7%).

There were no significant differences between treatment approach groups for gender, race, or ethnicity. However, the advanced PE intervention group was seven years younger than the anticoagulation monotherapy group. As shown in Table S4, the heart rate, respiratory rate, and shock index were lower in the anticoagulation monotherapy group. The mean systolic blood pressure was higher in the anticoagulation monotherapy group than in those who received advanced PE intervention. For PE risk factors, the anticoagulation monotherapy group had significantly greater proportions with dementia and known metastatic disease and significantly less with recent surgery, limb immobilization, nonmetastatic cancer, and hormonal replacement therapy. The advanced PE intervention group had significantly greater proportions with RV dilatation by imaging and elevated troponin (Table S4).

During the 1832 index PE hospitalizations, there were 94 deaths (5.1%) and 218 (11.9%) patients had one or more clinical deterioration events. Bivariate analyses (Table 1) show that death and clinical deterioration were significantly more common in those with advanced PE interventions than in those without advanced PE interventions (*p* < 0.001). Tables 2 and 3 show that death was strongly associated with clinical deterioration and major bleeding (*p* < 0.001).

Bivariate analysis of catheter-directed interventions versus systemic thrombolysis (Table 4) did not reveal significant differences in demographics, major bleeding events, and bleeding risk. There was significantly a greater immediacy of treatments with systemic thrombolysis vs CDI. A significantly greater proportion of patients with high-risk PE, clinical deterioration, and death were treated with systemic thrombolysis versus CDI. There was a trend towards significance for less major bleeding CDI (19/145 (13.1%)) versus systemic thrombolysis (33/154 (21.4%)). There was a significant variation in the type of advanced intervention approach chosen across centers. At one site, systemic thrombolysis was used ten times more than CDT, whereas, at three other clinical sites, CDT was used more than systemic thrombolysis.

**Figure 2:**
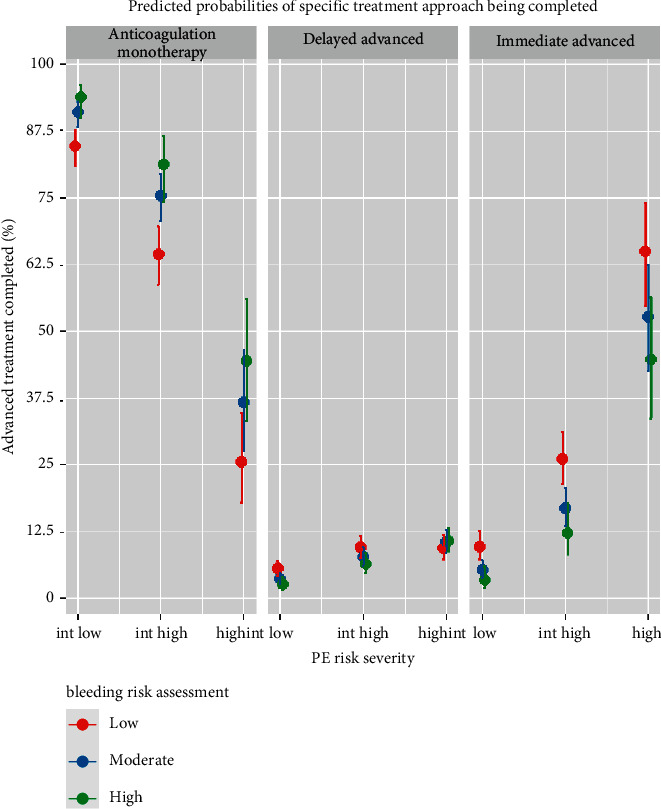
Predicted probability of each treatment approach.

Table 5 and Figure 2 demonstrate predicted probabilities of treatment approach based on each combination of PE severity and bleeding risk. For Table 5, we fit a regression model for the *ordinal outcome* of advanced PE intervention. There was no significant interaction effect between PE severity and bleeding risk assessment, so the model was fit with the main effects only. The left side of Table 5 shows that patients with high-risk PE were more than 2.5 times as likely to receive advanced PE intervention than those with intermediate-high-risk PE (odds ratio: 5.3 (4.2, 6.9) vs. 1.9 (1.6, 2.2), respectively). Advanced PE interventions were administered to 86 of the 139 high-risk PE patients (61.9%) compared with 195 of 707 intermediate-high-risk PE patients (27.6%) and 109 of 977 intermediate-low-risk PE patients (11.2%) (data not shown).

Table S5 shows that when the treatment approach was expressed as a *binary outcome* (advanced PE intervention vs anticoagulation monotherapy), high-risk PE had an OR of 14.3 (9.4, 21.9) vs 3.1 (2.4, 4.1) for those with intermediate-high-risk PE severity.

Table 5 also shows that patients with moderate and high bleeding risk were less likely to be treated with advanced PE interventions (OR: 0.71 (0.61–0.83) and 0.58 (0.46–0.73), respectively) than those with lower bleeding risk. While most patients with moderate to high bleeding risk received anticoagulation monotherapy, 73.5% of those with low bleeding risk also received anticoagulation monotherapy (no advanced PE intervention) (data not shown).

Figure 2 displays the predicted probability of each treatment approach based on PE severity and bleeding risk. The far-right panel shows that high-risk PE patients with low bleeding risk had slightly over 62% probability of receiving immediate advanced PE intervention (within 12 hours), while those with moderate bleeding risk had about 50% probability of immediate treatment, and those with the highest bleeding risk had about 40% probability. Conversely, the anticoagulation monotherapy panel shows that as bleeding risk increased for a fixed PE risk, the probability of receiving anticoagulation monotherapy increased.

Table S6 shows the exact predicted probabilities for each treatment approach based on the combination of PE severity and bleeding risk assessment. The odds of a more aggressive treatment increased as PE severity increased. Conversely, the odds of aggressive treatment decreased as bleeding risk increased. PE severity and bleeding risk assessment work additively when it comes to the overall OR or predicted probability of treatment approach.

#### 3.2.2. Secondary Outcomes

Table 6 provides a comparison of our secondary outcomes (clinical deterioration and major bleeding) by treatment approach. Overall, patients who received anticoagulation monotherapy were less likely to experience clinical deterioration than those who received advanced PE interventions. However, patients who received advanced PE interventions also received anticoagulation (i.e., the interventions are used in patients with signs of shock or those who suffer clinical deterioration despite a course of anticoagulation treatment). A greater proportion of those who received advanced PE intervention experienced major bleeding than those who did not.

Tables 2 and 3 display our secondary outcomes by the predictors of interest (PE severity, bleeding risk assessment, and PE severity/bleeding risk profile).  Table 2 shows that there were significant differences in each predictor of interest between the clinical deterioration outcome groups. Generally, those with a higher PE severity, higher bleeding risk, or higher combination of the two accounted for greater proportions of those with clinical deterioration. Of those with high-risk PE, 107 of 139 suffered clinical deterioration. In contrast, 76 of 707 intermediate-high-risk and 33 of 977 intermediate-low-risk PE patients suffered clinical deterioration. In the small low-risk group, 2 of 9 patients had clinical deterioration.

As shown in Table 3, 130 (7.1%) patients experienced one or more major bleeding events during the index PE hospitalization. There were significant differences between groups based on initial bleeding risk assessment. Major bleeding occurred in 21.5%, 50.4%, and 33.8% of those with high, moderate, and low bleeding risks, respectively.

There were some differences in patient characteristics between the secondary outcome groups.  Table S7 shows no difference in demographics between clinical deterioration groups (secondary outcome 1) but higher proportions with initial cardiac arrest and elevated RV by imaging and cardiac biomarkers in the clinical deterioration group than those without. Patients who had major bleeding (secondary outcome 2) were slightly younger than patients who did not (59.1 (16.3) and 63.1 (16.0) years, respectively). Vital signs were significantly different between secondary outcome groups with higher acuity vitals in the major bleeding group (higher respiratory rates, heart rates, hemodynamic collapse/cardiac arrest, and shock index, with lower systolic blood pressure and oxygen saturation).

Multivariate analyses showed that clinical deterioration was significantly more common in patients who had one or more advanced PE interventions but was more influenced by PE severity (high-risk OR: 56.33 and intermediate-high-risk OR: 2.61). Major bleeding was significantly associated with advanced PE interventions (OR: 3.34 for delayed and 5.23 for immediate) and high-risk PE (OR: 3.35). These results are displayed on the right side of Table 5. Logistic regression identified predictors of death (Table 7), expressed as odds ratios, immediate and delayed advanced interventions (5.5 (1.3 to 4.1) vs 4.3 (2.1 to 8.7)), and major bleeding (2.4 (1.3 to 4.1)).

## 4. Discussion

Despite solid evidence-based recommendations, advanced PE intervention was given to only 61.9% of those with high-risk PE than 27.6% and 11.2% of those with intermediate-high and intermediate-low-risk PE, respectively. Amongst the high-risk group, 81.4% with low bleeding risk received advanced PE intervention than 54.8% and 50% with moderate bleeding risk and high bleeding risk, respectively. High-risk PE was the highest predictor of receiving advanced PE intervention. Conversely, patients with a high bleeding risk had low odds of receiving advanced PE intervention. Death was strongly associated with clinical deterioration and major bleeding events. When advanced interventions were used, systemic thrombolysis was used more emergently than catheter-directed interventions in patients with high-risk PE severity.

Regarding outcomes, clinical deterioration occurred in 77.0% of patients with high-risk PE at presentation compared with 10.8% and 3.4% of intermediate-high-risk and intermediate-low-risk patients, respectively. Major bleeding occurred in 4.2% of anticoagulation monotherapy versus 17.6% of patients who received advanced PE interventions. Multivariate analyses showed that increased treatment aggression (beyond anticoagulation monotherapy) and increasing initial PE severity were associated with higher odds of clinical deterioration (including PE-related death) and major bleeding. The high bleeding risk was significantly associated with major bleeding, but not with clinical deterioration or PE-related death.

Not surprisingly, our data showed that high-risk PE patients were treated more aggressively and urgently than intermediate-risk PE patients. A closer look at the 139 patients with high-risk PE (Tables S3 and S6) shows that 59 (42.4%) had hemodynamic collapse/cardiac arrest upon presentation with increased proportions with advanced intervention, clinical deterioration, and major bleeding than the remaining 80 (57.8%) high-risk patients without initial hemodynamic collapse at presentation. Our results are similar to those reported by a PERT consortium study of 1442 high-risk PE patients [17]. In that study, high-risk PE patients were treated with advanced interventions more commonly than intermediate-risk PE (41.9% vs 30.2%), and high-risk PE patients with hemodynamic collapse had three times the mortality rate and more than double the rate of advanced intervention than high-risk PE patients without initial hemodynamic collapse.

Although patients who received anticoagulation monotherapy were less likely to experience clinical deterioration than those who received advanced PE intervention, one should not misinterpret this finding. There is likely no causal relationship between anticoagulation monotherapy and clinical deterioration. Anticoagulation monotherapy prevents the propagation of existing thrombus while the body's intrinsic thrombus lysis system works on dissolving the current thrombus over the course of days to weeks. In contrast, advanced PE interventions work to acutely remove thrombus and its burden. Ostensibly, the risk of anticoagulation monotherapy is a delayed reduction of thrombus burden and an increased risk of PE-provoked clinical deterioration. In our report, the use of advanced PE intervention was associated with increased odds of clinical deterioration and major bleeding. It is important to note that advanced PE interventions were coupled with anticoagulation during hospitalization. Although there was an increased risk of major bleeding when using an advanced PE intervention amongst the five main hospitals, EDs using CDTs over systemic thrombolysis had lower major bleeding complications than hospitals using systemic thrombolysis over CDTs.

Evidence has shown that systemic thrombolysis reduces mortality or hemodynamic instability but not enough in those with intermediate-risk PE to justify the increase in bleeding complications or when compared to anticoagulation monotherapy [18, 19]. Our study looked at treatment approaches including a subanalysis of the most common advanced interventions. In our study, major bleeding occurred in 13.1% of those treated with CDI versus 21.4% of those treated with systemic thrombolysis with a trend to significance (*p*=0.07). Treatment within 12 hours (immediate) occurred in 55.9% CDT versus 87.7% with systemic thrombolysis (*p* < 0.001). Any immediate advanced intervention was a strong independent predictor of death (OR: 5.5 (3.37–8.9)). In a meta-analysis (Planer et al.) of 44 studies with over 20,000 patients with intermediate- or high-risk PE, catheter-directed thrombolysis was associated with decreased risk of death and major bleeding compared to systemic thrombolysis while showing decreased death and no increase in major bleeding compared to anticoagulation monotherapy [20]. In our study, 61 of 1440 (4.2%) patients treated with anticoagulation monotherapy had a major bleeding event compared to 69 of 392 (17.6%) patients treated with any type of advanced intervention (Table 6). A meta-analysis of 12 studies by Ismayl et al. involving over 9000 patients with intermediate-risk PE showed no significant risk in bleeding events for those with catheter-directed thrombolysis intervention compared to those receiving anticoagulation monotherapy [6]. Another meta-analysis by Zhang et al. (some overlap with studies included in the Ismayl et al. meta-analysis) showed that catheter-directed thrombolysis has improved mortality with reduced intracranial bleeding risk when compared to systemic thrombolysis in intermediate-risk and high-risk PE with similar results in a subset analysis of patients with intermediate-risk PE [21]. Strategies to optimize the benefit and safety profile of advanced PE interventions include an investigation into the lowest doses of systemic thrombolytic agents that will be both effective and safe [22]. Several studies of FDA-approved CDTs have shown reductions in RV dilatation. Kucher et al. compared ultrasound-assisted catheter-directed thrombolysis with anticoagulation monotherapy in intermediate-risk patients. CDT showed a greater reduction in RV dilatation and no increase in bleeding complications within 90 days [23]. Single-arm studies of FDA-approved CDTs in patients with intermediate-risk PE have shown acute reductions in RV dilatation with low bleeding events or complications [24–26]. At the time of this report, the HI PEITHO trial was being conducted to address the safety and efficacy of advanced PE intervention versus anticoagulation in patients with intermediate-high-risk PE [27]. Ongoing studies address mechanical thrombectomy versus catheter-directed thrombolysis, with planned studies of mechanical thrombectomy versus anticoagulation monotherapy in patients with intermediate-risk PE [28].

There have been several recent reports on advanced PE interventions in hospitals with PERT programs. Most reports include management and mortality of PERT activations from the ED, medical and surgical floors, and ICUs. Our report focuses on the management and outcomes of PERT activations from EDs in a regional healthcare system with an established PERT program.

Several studies have looked for changes in patient management associated with the initiation of a PERT program. Several reports involve single centers and smaller sample sizes than our study. One study found the implementation of PERT increased the use of advanced PE interventions and improved outcomes [29]. Another study found no significant change in advanced PE intervention but improved 30-day mortality compared to the period before PERT was available at a single hospital [30]. Another study showed that initiation of a PERT led to substantial increases in the use of advanced interventions in high-risk PE from 30% to 92% and reduced time from diagnosis to advanced PE intervention from 12 hours to 3 hours [14]. Other single-center studies have shown that implementation of a PERT increased the use of advanced PE interventions for intermediate- and high-risk PE. In one report, advanced PE intervention use doubled from 15% to 32% [31]. Another study reported an increase in the use of ECMO (7.8%) and catheter-directed thrombolysis (46.3%) [32].

Unlike the cited studies above, our regional healthcare system had an established PE program (with a consensus-based treatment algorithm and PERT) for the duration of our registry database. Despite this, we found significant differences between the participating EDs in patient characteristics, primary outcome (treatment approach), and secondary outcomes (clinical deterioration and major bleeding). Overall, 21% of our ED PERT activations (patients with intermediate- and high-risk PE) received advanced PE intervention, and just 61.9% of high-risk PE patients received them. We also found differences in the use of advanced PE interventions within the three hospitals considered as PE referral sites in our system. They differed in the use of systemic thrombolysis versus CDTs. Thus, it seems clinical decision-making is independent of having a PERT. However, it was outside the scope of this study to determine if differences in treatment approach could be random or due to differences in patient characteristics, practice patterns of treating teams, or PERT availability at the different hospitals. Further investigation is needed to elucidate reasons for differences in treatment approach, which may include varying experience and risk-tolerance of clinicians, availability of more than one option of advanced PE interventions, hospital setting, staffing, and practice patterns. Future studies should include the composition and skill set of PERTs or multidisciplinary teams that decide if and when to use advanced PE interventions.

### 4.1. Limitations

Our report has several limitations. First, we did not prospectively determine the rationale for decisions to consider anticoagulation versus advanced PE interventions for each patient. Studying the factors involved in the clinical decision-making of a large clinical team per patient and determining available resources for a large sample of patients were beyond the scope of this hypothesis-generating study. Our investigator team anecdotally noted day-to-day and hospital-to-hospital variabilities in the composition of our PERT. Such variability has been reported by a national multicenter analysis of 475 unique PERT activations [33]. It is possible that the varying PERT composition influenced decisions and agreement about treatment approaches on a case-by-case basis. It would be helpful to have observational and/or qualitative studies that report on criteria of importance to clinical decision-making in intermediate/high-risk PE.

Second, physician and institution experience and expertise in advanced PE interventions at our regional healthcare ED may not be generalizable. Some facilities within our healthcare system were recommended destinations for higher acuity PE patients, whereas other hospitals performed more CDTs.

Third, advanced PE interventions differ in availability and use of resources. Systemic thrombolysis is widely available and can be easily administered at the bedside. In contrast, CDT requires special rooms, capital, and procedural skill sets. We did not include a report on the use of CDT, the more resource-intensive advanced treatment.

Fourth, we used ESC criteria for defining high-risk PE severity in our analyses for this report. A recent PERT report, which stratified high-risk severity patients into subgroups with or without hemodynamic collapse/cardiac arrest, noted significant differences in outcomes of advanced treatment and mortality [17]. In our study, bivariate analyses show that the presence of hemodynamic collapse was significantly higher in those primary and secondary outcomes. Although there was an opportunity to include high-risk with and without hemodynamic instability/cardiac arrest, this high-risk PE subgroup was not a part of the ESC risk assignments used in our study design. Further granularity is possible in characterizing the association between high-risk PE severity subgroups and our primary and secondary outcomes.

Finally, we used the start time of the electronic order entry of medication or the start time of procedural interventions to determine the timing of advanced PE intervention. The time of completion of advanced PE interventions would be better for completion of advanced PE interventions that take longer to perform.

## 5. Conclusions

Ideally, advanced PE interventions should be widely available, effective, and safe for patients with intermediate-high and high-risk PE. In our regional healthcare system, we uncovered considerable variation in practice. In real-world circumstances, the use of advanced PE interventions after PERT activations did not fully follow evidence-based recommendations and in that close to 40% of high-risk PE patients did not receive any of the current options of advanced PE intervention, while over a quarter of intermediate-high-risk patients did. We also noted differences in the type of advanced PE interventions used between our PE referral sites. However, the rationale for the treatment approach was not explored. Any association between treatment approach and clinical deterioration in this study was a product of the appropriateness of treatment based on PE severity and bleeding risk profile.

The association with our other secondary outcome (major bleeding complications) was more apparent: a greater proportion of those who received advanced intervention experienced major bleeding than those who did not. Although advanced interventions were associated with high-acuity patients experiencing death, clinical deterioration, and major bleeding, there was a trend towards less bleeding with catheter-directed interventions versus systemic thrombolysis. Our findings underscore the importance of a careful selection of advanced interventions that provide noninferior or improved efficacy over systemic thrombolysis to limit major bleeding complications among patients with high-risk and intermediate-high-risk PE.

## Figures and Tables

**Table 1:** Patient characteristics and outcomes by primary outcome (treatment approach)^*∗*^.

Characteristics	Overall *N* = 1832	Anticoagulation monotherapy, *N* = 1440	Delayed advanced PE intervention (>12 hours) *N* = 113	Immediate advanced PE intervention (within 12 hours) *N* = 279	*P* value (difference by ANOVA or chi-square)
*Demographics*					
Mean age, years (SD)	62.8 (16.0)	64.3 (15.9)	57.3 (14.8)	57.5 (15.5)	<0.001
Gender: cis male, *n* (%)	890 (48.6%)	690 (47.9%)	61.0 (54.0%)	139 (49.8%)	0.438
Gender: cis female, *n* (%)	942 (51.4%)	750 (52.1%)	52.0 (46.0%)	140 (50.2%)	

*Race, n (%)*					
Caucasian	1125 (61.4%)	891 (61.9%)	70 (61.9%	164 (58.8%)	0.713
African-American	642 (35.0%)	492 (34.2%)	40 (35.4%)	110 (39.4%)	
American-Indian/Alaskan	16 (0.9%)	15 (1.0%)	1 (0.9%)	0 (0%)	
Asian	5 (0.3%)	4 (0.3%)	0 (0%)	1 (0.4%)	
Pacific Islander	1 (0.1%)	1 (0.1%)	0 (0%)	0 (0%)	
Other	9 (0.5%)	9 (0.6%)	0 (0%)	0 (0%)	
Missing	34 (1.9%)	28 (1.9%)	2 (1.8%)	4 (1.4%)	

*Ethnicity, n (%)*					
Hispanic	43 (2.3%)	35 (2.4%)	1 (0.9%)	7 (2.5%)	0.799
Non-Hispanic	1704 (93.0%)	1336 (92.8%)	106 (93.8%)	262 (93.9%)	
Other	85 (4.6%)	69 (4.8%)	6 (5.3%)	10 (3.6%)	

*Initially eligible for advanced PE intervention, n (%)*					
Yes	855 (46.7%)	564 (39.2%)	71 (62.8%)	220 (78.9%)	<0.001
No	977 (53.3%)	876 (60.8%)	42 (37.2%)	59 (21.1%)	

*PE severity at presentation* ^†^ *n (%)*					
High risk	139 (7.6%)	53 (3.7%)	7 (6.2%)	79 (28.3%)	<0.001
Intermediate-high risk	707 (38.6%)	512 (35.6%)	62 (54.9%)	133 (47.7%)	
Intermediate-low risk	977 (53.3%)	868 (60.3%)	44 (38.9%)	65 (23.3%)	
Low risk	9 (0.5%)	7 (0.5%)	0 (0%)	2 (0.7%)	

*Bleeding risk assessment, n (%)*					
High	248 (13.5%)	200 (13.9%)	21 (18.6%)	27 (9.7%)	<0.001
Moderate	915 (49.9%)	748 (51.9%)	42 (37.2%)	125 (44.8%)	
Low	669 (36.5%)	492 (34.2%)	50 (44.2%)	127 (45.5%)	

*Initial PE severity/bleeding risk profile, n (%)*					
High PE/high bleeding risk	34 (1.9%)	17 (1.2%)	4 (3.5%)	13 (4.7%)	<0.001
High PE/moderate bleeding risk	62 (3.4%)	28 (1.9%)	1 (0.9%)	33 (11.8%)	
High PE/low bleeding risk	43 (2.3%)	8 (0.6%)	2 (1.8%)	33 (11.8%)	
Intermediate-high PE/high bleeding risk	113 (6.2%)	90 (6.3%)	12 (10.6%)	11 (3.9%)	
Intermediate-high PE/moderate bleeding risk	355 (19.4%)	267 (18.5%)	22 (19.5%)	66 (23.7%)	
Intermediate-high PE/low bleeding risk	239 (13.0%)	155 (10.8%)	28 (24.8%)	56 (20.1%)	
Intermediate-low PE/high bleeding risk	101 (5.5%)	93 (6.5%)	5 (4.4%)	3 (1.1%)	
Intermediate-low PE/moderate bleeding risk	494 (27.0%)	450 (31.3%)	19 (16.8%)	25 (9.0%)	
Intermediate-low PE/low bleeding risk	391 (21.3%)	332 (23.1%)	20 (17.7%)	39 (14.0%)	

*Clinical outcomes*					
Death	39 (2.7%)	12 (10.6%)	43 (15.4%)	94 (5.1%)	<0.001
Clinical deterioration	98 (6.8%)	18 (15.9%)	102 (36.6%)	218 (11.9%)	<0.001

^*∗*^The *percentages* within each cell were calculated using the *N* in the *column header* for that cell (e.g., of all patients who received anticoagulation monotherapy, 53/1440 (3.7%) were classified as high-risk PE at ED presentation. Conversely, all 139 (38.1%) high-risk PE patients in this study had anticoagulation monotherapy). ^†^We found the following proportions for each criterion used to determine PE severity: 81% had a RV : LV ratio of 1.0 or greater as determined by CT, 22% had RV dilatation by echocardiography, 3.2% arrived in cardiac arrest, 5.0% required vasopressor support at presentation, 5.6% had sustained hypotension, 5.2% had episodic hypotension, 17.8% had a sustained elevated shock index, 36.2% had hypoxia with respiratory distress at rest, 68% had elevated troponin, and 57.2% had elevated brain natriuretic peptide levels.

**Table 2:** Secondary outcome 1 (clinical deterioration) by predictors of interest and death^*∗*^.

Predictors of interest	No clinical deterioration (*N* = 1614)	Clinical deterioration (*N* = 218)	Overall (*N* = 1832)	Difference *t*-test or chi-square, *p* value

*PE severity at presentation, N (%)*				
High risk	32 (2.0%)	107 (49.1%)	139 (7.6%)	<0.001
Intermediate-high risk	631 (39.1%)	76 (34.9%)	707 (38.6%)	
Intermediate-low risk	944 (58.5%)	33 (15.1%)	977 (53.3%)	
Low risk	7 (0.4%)	2 (0.9%)	9 (0.5%)	
*Bleeding risk assessment, N (%)*				
High	203 (12.6%)	45 (20.6%)	248 (13.5%)	0.006
Moderate	812 (50.3%)	103 (47.2%)	915 (49.9%)	
Low	599 (37.1%)	70 (32.1%)	669 (36.5%)	

PE severity and bleeding risk profile, *N* (%)	No clinical deterioration (*N* = 1614)	Clinical deterioration (*N* = 218)	Overall (*N* = 1832)	

High PE/high bleeding risk	15 (0.9%)	19 (8.7%)	34 (1.8%)	<0.001
High PE/moderate bleeding risk	12 (0.7%)	50 (22.9%)	62 (3.4%)	
High PE/low bleeding risk	5 (0.3%)	38 (17.4%)	43 (2.3%)	
Intermediate-high PE/high bleeding risk	93 (5.8%)	20 (9.2%)	113 (6.2%)	
Intermediate-high PE/moderate bleeding risk	319 (19.8%)	36 (16.5%)	355 (19.4%)	
Intermediate-high PE/low bleeding risk	219 (13.6%)	20 (9.2%)	239 (13.0%)	
Intermediate-low PE/high bleeding risk	95 (5.9%)	6 (2.8%)	101 (5.5%)	
Intermediate-low PE/moderate bleeding risk	478 (29.6%)	16 (7.3%)	494 (27.0%)	
Intermediate-low PE/low bleeding risk	378 (23.4%)	13 (6.0%)	391 (21.3%)	
*Outcome*				
Death	10 (0.6%)	84 (38.5%)	94 (5.1%)	<0.001

^*∗*^The *percentages* within each cell were calculated using the *N* in the *column header* for that cell.

**Table 3:** Secondary outcome 2 (major bleeding) by predictors and outcomes of interest^*∗*^.

	No bleeding (*N* = 1702)	Major bleeding (*N* = 130)	Overall (*N* = 1832)	Difference t-test or chi-square, *p* value
*PE severity at presentation, N (%)*				
High risk	102 (6.0%)	37 (28.5%)	139 (7.6%)	<0.001
Intermediate-high risk	661 (38.8%)	46 (35.4%)	707 (38.6%)	
Intermediate-low risk	931 (54.7%)	46 (35.4%)	977 (53.3%)	
Low risk	8 (0.5%)	1 (0.8%)	9 (0.5%)	

*Bleeding risk assessment, N (%)*				
High	220 (12.9%)	28 (21.5%)	248 (13.5%)	0.028
Moderate	857 (50.4%)	58 (44.6%)	915 (49.9%)	
Low	625 (36.7%)	44 (33.8%)	669 (36.5%)	

*PE severity/bleeding risk assessment profile, N (%)*				
High-risk PE/high bleeding risk	24.0 (1.4%)	10.0 (7.7%)	34 (1.9%)	<0.001
High-risk PE/moderate bleeding risk	52 (3.1%)	10 (7.7%)	62 (3.4%)	
High-risk PE/low bleed risk	26 (1.5%)	17 (13.1%)	43 (2.3%)	
Intermediate-high PE/high bleeding risk	99 (5.8%)	14 (10.8%)	113 (6.2%)	
Intermediate-high PE/moderate bleeding risk	334 (19.6%)	21 (16.2%)	355 (19.4%)	
Intermediate-high PE/low bleeding risk	228 (13.4%)	11 (8.5%)	239 (13.0%)	
Intermediate-low PE/high bleeding risk	97 (5.7%)	4 (3.1%)	101 (5.5%)	
Intermediate-low PE/moderate bleeding risk	468 (27.5%)	26 (20.0%)	494 (27.0%)	
Intermediate-low PE/low bleeding risk	374 (22.0%)	17 (13.1%)	391 (21.3%)	

*Outcomes*				
Death	73 (4.3%)	21 (16.2%)	94 (5.1%)	<0.001
Clinical deterioration	160 (9.4%)	58 (44.6%)	218 (11.9%)	<0.001

^*∗*^The *percenta*ges within each cell were calculated by using the *N* in the *column header* for that cell.

**Table 4:** Bivariate analysis grouped by two main advanced interventions (catheter-directed interventions versus systemic thrombolysis)

	Catheter-directed intervention (*N* = 145)	Systemic thrombolysis (*N* = 154)	Overall (*N* = 299)	*P* value
*Age*				
Mean (SD), years	55.8 (15.0)	55.4 (15.1)	55.6 (15.0)	0.816

*Race*				
White	79 (54.5%)	90 (58.4%)	169 (56.5%)	0.714
Black	62 (42.8%)	61 (39.6%)	123 (41.1%)	
American-Indian/Alaskan	1 (0.7%)	0 (0%)	1 (0.3%)	
Asian	1 (0.7%)	0 (0%)	1 (0.3%)	
Unknown	2 (1.4%)	3 (1.9%)	5 (1.7%)	

*Gender*				
Female	71 (49.0%)	77 (50.0%)	148 (49.5%)	0.908
Male	74 (51.0%)	77 (50.0%)	151 (50.5%)	

*Ethnicity*				
Hispanic	2 (1.4%)	4 (2.6%)	6 (2.0%)	0.376
Non-Hispanic	135 (93.1%)	146 (94.8%)	281 (94.0%)	
Unknown	8 (5.5%)	4 (2.6%)	12 (4.0%)	

*Advanced treatment timing*				
Yes (delayed >12 hrs later)	64 (44.1%)	18 (11.7%)	82 (27.4%)	<0.001
Yes (within 12 hrs)	81 (55.9%)	135 (87.7%)	216 (72.2%)	

*Major bleeding*				
No	126 (86.9%)	121 (78.6%)	247 (82.6%)	0.0673
Yes	19 (13.1%)	33 (21.4%)	52 (17.4%)	

*Clinical deterioration*				
CD	18 (12.4%)	72 (46.8%)	90 (30.1%)	<0.001
No CD	127 (87.6%)	82 (53.2%)	209 (69.9%)	

*Death*				
No	139 (95.9%)	124 (80.5%)	263 (88.0%)	<0.001
Yes	6 (4.1%)	30 (19.5%)	36 (12.0%)	

*PE severity at presentation*				
High risk	9 (6.2%)	57 (37.0%)	66 (22.1%)	<0.001
Intermediate-high risk	81 (55.9%)	76 (49.4%)	157 (52.5%)	
Intermediate-low risk	55 (37.9%)	20 (13.0%)	75 (25.1%)	
Low risk	0 (0%)	1 (0.6%)	1 (0.3%)	

*Bleeding risk*				
High	14 (9.7%)	14 (9.1%)	28 (9.4%)	0.904
Moderate	60 (41.4%)	68 (44.2%)	128 (42.8%)	
Low	71 (49.0%)	72 (46.8%)	143 (47.8%)	

*PE severity/bleeding risk profile*				
High-risk PE/high bleeding risk	2 (1.4%)	8 (5.2%)	10 (3.3%)	<0.001
High-risk PE/moderate bleeding risk	3 (2.1%)	27 (17.5%)	30 (10.0%)	
High-risk PE/low bleeding risk	4 (2.8%)	22 (14.3%)	26 (8.7%)	
Intermediate-high-risk PE/high bleeding risk	11 (7.6%)	5 (3.2%)	16 (5.4%)	
Intermediate-high PE/moderate bleeding risk	35 (24.1%)	34 (22.1%)	69 (23.1%)	
Intermediate-high PE/low bleeding risk	35 (24.1%)	37 (24.0%)	72 (24.1%)	
Intermediate-low PE/high bleeding risk	1 (0.7%)	1 (0.6%)	2 (0.7%)	
Intermediate-low PE/moderate bleeding risk	22 (15.2%)	6 (3.9%)	28 (9.4%)	
Intermediate-low PE/low bleeding risk	32 (22.1%)	14 (9.1%)	46 (15.4%)	

*Clinical site*				
AH Carolinas Medical Center	8 (5.5%)	89 (57.8%)	97 (32.4%)	<0.001
AH Cabarrus	34 (23.4%)	18 (11.7%)	52 (17.4%)	
AH Mercy	1 (0.7%)	1 (0.6%)	2 (0.7%)	
AH Pineville	68 (46.9%)	19 (12.3%)	87 (29.1%)	
AH Union	3 (2.1%)	7 (4.5%)	10 (3.3%)	
AH University City	28 (19.3%)	3 (1.9%)	31 (10.4%)	
CHS Blue Ridge/Morganton	1 (0.7%)	2 (1.3%)	3 (1.0%)	
AH Cleveland	0 (0%)	9 (5.8%)	9 (3.0%)	
AH Kings Mountain	0 (0%)	1 (0.6%)	1 (0.3%)	
AH Stanly	0 (0%)	1 (0.6%)	1 (0.3%)	
Missing	2 (1.4%)	4 (2.6%)	6 (2.0%)	

**Table 5:** Multivariable analyses of primary (ordinal) and secondary outcomes^*∗*^.

Predictors	Primary outcome (ordinal)			Secondary outcomes					
	Advanced PE intervention			Clinical deterioration			Major bleeding		
	Odds ratio	Confidence interval	*P* value	Odds ratios	Confidence interval	*P* value	Odds ratio	Confidence interval	*P* value
(Intercept)				0.02	0.02–0.04	<0.001	0.03	0.02–0.05	<0.001
Intervention (delayed >12 hrs later)	NA	NA	NA	2.66	1.41–5.01	0.003	3.34	1.79–6.21	0.001
Intervention (within 12 hrs)	NA	NA	NA	3.55	2.31–5.44	<0.001	5.23	3.50–7.81	<0.001
Moderate bleeding risk	0.71	0.61–0.83	<0.001	1.20	0.79–1.82	0.388	1.09	0.71–1.67	0.701
High bleeding risk	0.58	0.46–0.73	<0.001	1.45	0.83–2.51	0.189	1.90	1.10–3.24	0.019
Intermediate-high risk PE	1.90	1.64–2.20	<0.001	2.61	1.68–4.04	<0.001	1.02	0.66–1.59	0.914
High-risk PE	5.32	4.17–6.79	<0.001	56.33	31.99–99.21	<0.001	3.35	1.92–5.80	<0.001
Observations^†^	1823			1823					
R2 nagelkerke 0.174				R2 tjur 0.366	R2 tjur	0.081			

^*∗*^Ordinal regression for primary outcome (treatment approach); multivariable analysis for binary secondary outcomes (clinical deterioration and major bleeding). *Note.* NA = not applicable, PE = pulmonary embolism. ^†^The number of observations is less than the study sample size due to missing data for some patients.

**Table 6:** Comparison of secondary outcomes by treatment approach^*∗*^.

Treatment approach	Secondary outcome 1: clinical deterioration			Overall (*N* = 1832)	Difference *t*-test or chi-square, *p* value
	No clinical deterioration (*N* = 1614)		Clinical deterioration (*N* = 218)		

Anticoagulation monotherapy	1342 (83.1%)		98 (45.0%)	1440 (78.6%)	<0.001
Delayed advanced PE intervention	95 (5.9%)		18 (8.3%)	113 (6.2%)	
Immediate advanced PE intervention	177 (11.0%)		102 (46.8%)	279 (15.2%)	

Treatment approach	Secondary outcome 2: major bleeding			Overall (*N* = 1832)	Difference *t*-test or chi-square, *p* value
	No bleeding (*N* = 1702)	Major bleeding (*N* = 130)			

Anticoagulation monotherapy	1379 (81.0%)	61 (46.9%)		1440 (78.6%)	<0.001
Delayed advanced PE intervention	99 (5.8%)	14 (10.8%)		113 (6.2%)	
Immediate advanced PE intervention	224 (13.2%)	55 (42.3%)		279 (15.2%)	

Breakout: advanced PE intervention (regardless of timing) by type^†^	Secondary outcome 2: major bleeding			Overall (*N* = 1832)	Difference *t*-test or chi-square, *p* value
	No bleeding (*N* = 1702)	Major bleeding (*N* = 130)			

Systemic thrombolysis	121 (7.1%)	33 (25.4%)		154 (8.4%)	<0.001
Catheter-directed treatment	127 (7.5%)	20 (15.4%)		147 (8.0%)	0.00366
Surgical embolectomy	2 (0.1%)	7 (5.4%)		9 (0.5%)	<0.001
ECMO	0 (0%)	10 (7.7%)		10 (0.5%)	<0.001
Other types of advanced intervention	3 (0.2%)	1 (0.8%)		4 (0.2%)	0.255

^*∗*^The *percentages* within each cell were calculated using the *N* in the *column header* for that cell. ^†^Some patients had more than one type of advanced PE intervention.

**Table 7:** Multivariate logistic regression analysis of predictors of death.

Predictors	Odds of PE-related death		
	Odds ratios	CI	*P* value
Intercept	0.02	0.02–0.04	<0.001
Delayed advanced intervention	4.31	2.14–8.69	<0.001
Immediate advanced intervention	5.48	3.37–8.92	<0.001
Major bleeding	2.34	1.32–4.14	0.004

*Random effects*			
*σ* ^2^	3.29		
*τ*_00hosp_	0.16		
ICC	0.05		
Number_clinicalsite_	6		
Observations	1767		
Marginal *R*^2^/conditional *R*^2^	0.141/0.181		

ICC = intraclass correlation; PE = pulmonary embolism.

## Data Availability

The data used to support the findings of the study are available from the corresponding author upon request.

## Supplementary Materials

Supplementary Materials Table S1: classification criteria for pulmonary embolism severity and bleeding risk assessment. Table S2: advanced PE treatment options based on PE severity and bleeding risk at presentation. Table S3: patient characteristics and outcomes grouped by hospital emergency departments. Table S4: supplemental data on patient characteristics by primary outcome (treatment approach). Table S5: multivariate analyses of treatment approach expressed as binary outcome (advanced PE intervention vs. anticoagulation monotherapy). Table S6: probability of treatment completed on PE severity (intermediate/high-risk) and bleeding risk at presentation expressed as percentages with 95% confidence intervals. Table S7: patient characteristics by secondary outcomes.
