# Supplementary material for: Comparison of Treatment Approaches and Subsequent Outcomes within a Pulmonary Embolism Response Team Registry
Source: Crit Care Res Pract. 2024 Mar 22;2024:5590805. doi: 10.1155/2024/5590805 (PMC10980543; doi:10.1155/2024/5590805)
Supplement: Supplementary Materials — Table S1: classification criteria for pulmonary embolism severity and bleeding risk assessment. Table S2: advanced PE treatment options based on PE severity and bleeding risk at presentation. Table S3: patient characteristics and outcomes grouped by hospital emergency departments. Table S4: supplemental data on patient characteristics by primary outcome (treatment approach). Table S5: multivariate analyses of treatment approach expressed as binary outcome (advanced PE intervention vs. anticoagulation monotherapy). Table S6: probability of treatment completed on PE severity (intermediate/high-risk) and bleeding risk at presentation expressed as percentages with 95% confidence intervals. Table S7: patient characteristics by secondary outcomes. [file 5590805.f1.zip › Table S1.docx]

*Table S1: Classification criteria for pulmonary embolism severity and bleeding risk assessment.*

| *PE severity* | *Definition and criteria* |  | *Bleeding Risk Assessment* | *Definition and criteria* |
| --- | --- | --- | --- | --- |
| *High-risk* | Presence of right ventricular (RV) dilatation in the absence of primary unstable dysrhythmia or other cause, such as severe sepsis (although conditions may co-exist)  one or more signs of RV dilatation or dysfunction: RV diameter greater than left ventricular (LV) diameter on CT or echocardiography plus   - sustained hypotension (systolic blood pressure less than 90 mmHg for > 15 minutes), - need for vasopressors at presentation, or - cardiac arrest. |  | *High bleeding risk* | If any of the following historical criteria were present:   - gastrointestinal bleed within 30 days; - life threatening hemorrhage; - head trauma causing loss of consciousness within previous seven days; - history of hemorrhage stroke; - ischemic stroke within one year; - intracranial metastasis or - platelet count < 50,000/uL; - liver failure with international normalized ratio > 1.7; - surgery that required opening the chest cavity, peritoneum, skull, or spinal canal within previous 14 days; - pregnancy; and - large pericardial effusion |
| *Intermediate-high risk* | one or more signs of RV dilatation or dysfunction: RV diameter greater than left ventricular (LV) diameter on CT or echocardiography, or elevated cardiac biomarkers (troponin or natriuretic peptide) AND   - episodic hypotension (< 90 mmHg) OR - shock index consistently > 1.0 or - arterial oxygen saturation < 92% (on room air) with distress |  | *Moderate bleeding risk* | If one or more of the following historical criteria were present:   - age > 65 years; recent surgery (> 14 days but < 60 days); - recent transient ischemic attack or stroke-like symptoms; - any prior gastrointestinal bleeding; - current use of thienopyridine, direct thrombin inhibitors, or factor Xa inhibitors; - any metastatic cancer; - recent tongue bite; - recent fracture; - recent fall with head strike; - history of hematuria or frequent nosebleeds; and - severe uncontrolled systolic blood pressure > 180 mmHg or diastolic blood pressure >110 mmHg on initial presentation. |
| *Intermediate-low risk* | + RV dilatation signs but none of the criteria listed for high- or intermediate-high risk |  |  |  |
| *Low-risk* | *Absence of RV dilatation, normal and stable vital signs:* Provider concerned by elevated cardiac biomarkers or concerning deep vein thrombosis (DVT) |  | *Low risk* | None of the criteria for high or moderate bleeding risk applied to them |

CT= computed tomography; LV= left ventricle; PE= pulmonary embolism; RV= right ventricle
