# Supplementary material for: Comparison of Treatment Approaches and Subsequent Outcomes within a Pulmonary Embolism Response Team Registry
Source: Crit Care Res Pract. 2024 Mar 22;2024:5590805. doi: 10.1155/2024/5590805 (PMC10980543; doi:10.1155/2024/5590805)
Supplement: Supplementary Materials — Table S1: classification criteria for pulmonary embolism severity and bleeding risk assessment. Table S2: advanced PE treatment options based on PE severity and bleeding risk at presentation. Table S3: patient characteristics and outcomes grouped by hospital emergency departments. Table S4: supplemental data on patient characteristics by primary outcome (treatment approach). Table S5: multivariate analyses of treatment approach expressed as binary outcome (advanced PE intervention vs. anticoagulation monotherapy). Table S6: probability of treatment completed on PE severity (intermediate/high-risk) and bleeding risk at presentation expressed as percentages with 95% confidence intervals. Table S7: patient characteristics by secondary outcomes. [file 5590805.f1.zip › Table S2.docx]

| **Table S2:** Advanced PE treatment options based on PE severity and bleeding risk at presentation* | | | |
| --- | --- | --- | --- |
|  | Bleeding Risk Assignment at presentation^†^ | | |
| PE severity  at ED presentation | High  bleeding risk | Moderate  bleeding risk | Low  bleeding risk |
| High-risk PE | Catheter-directed treatment or VA ECMO or embolectomy | Systemic thrombolysis 1st option & if shock persists  Consider: catheter- directed treatment or embolectomy | Systemic thrombolysis as 1st option & if shock persists consider: catheter-directed treatment or embolectomy |
| Intermediate-high risk PE | Anticoagulation monotherapy or catheter-directed embolectomy | Catheter-directed treatment or reduced dose thrombolysis | Systemic thrombolysis or catheter-directed treatment |
| Intermediate low risk | Anticoagulation monotherapy; watch and wait; IVC filter | Anticoagulation monotherapy | Anticoagulation monotherapy |
| Low risk | IVC filter | Anticoagulation monotherapy | Anticoagulation monotherapy |

* Abbreviations: AC = anticoagulation monotherapy, IVC = Inferior vena cava, PE= pulmonary embolism; VA ECMO = veno-arterial extracorporeal membrane oxygenation, ST = systemic thrombolysis

^†^ Shaded boxes denote ineligibility for advanced PE interventions at presentation. Relative contraindications to advanced pulmonary embolism interventions: high bleeding risk, do-not-resuscitate (DNR) status, and physician discretion.
