# Supplementary material for: Comparison of Treatment Approaches and Subsequent Outcomes within a Pulmonary Embolism Response Team Registry
Source: Crit Care Res Pract. 2024 Mar 22;2024:5590805. doi: 10.1155/2024/5590805 (PMC10980543; doi:10.1155/2024/5590805)
Supplement: Supplementary Materials — Table S1: classification criteria for pulmonary embolism severity and bleeding risk assessment. Table S2: advanced PE treatment options based on PE severity and bleeding risk at presentation. Table S3: patient characteristics and outcomes grouped by hospital emergency departments. Table S4: supplemental data on patient characteristics by primary outcome (treatment approach). Table S5: multivariate analyses of treatment approach expressed as binary outcome (advanced PE intervention vs. anticoagulation monotherapy). Table S6: probability of treatment completed on PE severity (intermediate/high-risk) and bleeding risk at presentation expressed as percentages with 95% confidence intervals. Table S7: patient characteristics by secondary outcomes. [file 5590805.f1.zip › Table S3.docx]

**Table S3:** Patient characteristics and outcomes grouped by hospital emergency departments

|  | Hospital A  (N=513) | Hospital B  (N=329) | Hospital C  (N=108) | Other Hospitals*  (N=204) | Hospital D  (N=485) | Hospital E  (N=137) | Overall  (N=1832) | p-value |
| --- | --- | --- | --- | --- | --- | --- | --- | --- |
| **Age, years** |  |  |  |  |  |  |  |  |
| Mean (SD) | 60.7 (15.9) | 64.9 (15.7) | 65.6 (13.6) | 64.0 (15.8) | 63.8 (16.3) | 60.2 (16.5) | 62.8 (16.0) | <0.001 |
| **Race** |  |  |  |  |  |  |  | <0.001 |
| White | 268 (52.2%) | 244 (74.2%) | 82 (75.9%) | 132 (64.7%) | 321 (66.2%) | 47 (34.3%) | 1125 (61.4%) |  |
| Black | 223 (43.5%) | 78 (23.7%) | 24 (22.2%) | 64 (31.4%) | 144 (29.7%) | 86 (62.8%) | 642 (35.0%) |  |
| Native American/  Alaskan | 7 (1.4%) | 0 (0%) | 1 (0.9%) | 0 (0%) | 6 (1.2%) | 1 (0.7%) | 16 (0.9%) |  |
| Asian | 1 (0.2%) | 1 (0.3%) | 0 (0%) | 0 (0%) | 2 (0.4%) | 1 (0.7%) | 5 (0.3%) |  |
| Pacific  Islander | 0 (0%) | 0 (0%) | 0 (0%) | 1 (0.5%) | 0 (0%) | 0 (0%) | 1 (0.1%) |  |
| Other | 1 (0.2%) | 3 (0.9%) | 1 .(0.9%) | 1 (0.5%) | 3 (0.6%) | 0 (0%) | 9 (0.5%) |  |
| Unknown | 13 (2.5%) | 3 (0.9%) | 0 (0%) | 6 (2.9%) | 9 (1.9%) | 2 (1.5%) | 34 (1.9%) |  |
| **Gender, N (%)** |  |  |  |  |  |  |  | 0.97 |
| Female | 261 (50.9%) | 174 (52.9%) | 53 (49.1%) | 107 (52.5%) | 254 (52.4%) | 68 (49.6%) | 942 (51.4%) |  |
| Male | 252 (49.1%) | 155 (47.1%) | 55 (50.9%) | 97 (47.5%) | 231 (47.6%) | 69 (50.4%) | 890 (48.6%) |  |
|  |  |  |  |  |  |  |  |  |
| **Hispanic ethnicity** | 11 (2.1%) | 9 (2.7%) | 0 (0%) | 4 (2.0%) | 14 (2.9%) | 3 (2.2%) | 43 (2.3%) | 0.753 |
| **Treatment provided, N (%)** |  |  |  |  |  |  |  |  |
| Anticoagulation monotherapy | 375 (73.1%) | 263 (79.9%) | 96 (88.9%) | 185 (90.7%) | 374 (77.1%) | 97 (70.8%) | 1440 (78.6%) | <0.001 |
| Advanced intervention (delayed > 12 hrs later) | 26 (5.1%) | 18 (5.5%) | 3 (2.8%) | 8 (3.9%) | 42 (8.7%) | 14 (10.2%) | 113 (6.2%) |  |
| Advanced intervention (within 12 hrs) | 112 (21.8%) | 48 (14.6%) | 9 (8.3%) | 11 (5.4%) | 69 (14.2%) | 26 (19.0%) | 279 (15.2%) |  |
|  |  |  |  |  |  |  |  |  |
| **Systemic thrombolysis, N (%)** | 89 (17.3%) | 18 (5.5%) | 9 (8.3%) | 12 (5.9%) | 19 (3.9%) | 3 (2.2%) | 154 (8.4%) | <0.001 |
|  |  |  |  |  |  |  |  |  |
| **Catheter- directed intervention, N (%)** | 8 (1.6%) | 36 (10.9%) | 0 (0%) | 5 (2.5%) | 68 (14.0%) | 28 (20.4%) | 147 (8.0%) | <0.001 |
|  |  |  |  |  |  |  |  |  |
| **Surgical embolectomy, N (%)** | 7 (1.4%) | 1 (0.3%) | 0 (0%) | 0 (0%) | 0 (0%) | 1 (0.7%) | 9 (0.5%) | 0.0405 |
| **ECMO, N (%)** | 9 (1.8%) | 1 (0.3%) | 0 (0%) | 0 (0%) | 0 (0%) | 0 (0%) | 10 (0.5%) | 0.005 |
| **RV:LV > 1.0 by CT** | 404 (78.8%) | 277 (84.2%) | 88.0 (81.5%) | 163 (79.9%) | 394 (81.2%) | 115 (83.9%) | 1484 (81.0%) | 0.445 |
|  |  |  |  |  |  |  |  |  |
| **RV dilatation by echo** | 169 (32.9%) | 65 (19.8%) | 20 (18.5%) | 35 (17.2%) | 88 (18.1%) | 23 (16.8%) | 403 (22.0%) | <0.001 |
| **Cardiac Arrest at initial presentation** | 34 (6.6%) | 6 (1.8%) | 3 (2.8%) | 0 (0%) | 12 (2.5%) | 3 (2.2%) | 59 (3.2%) | <0.001 |
|  |  |  |  |  |  |  |  |  |
| **Vasopressors** | 53 (10.3%) | 16 (4.9%) | 7 (6.5%) | 5 (2.5%) | 10 (2.1%) | 1(0.7%) | 92 (5.0%) | <0.001 |
| Sustained hypotension | 48 (9.4%) | 23 (7.0%) | 7 (6.5%) | 4 (2.0%) | 16 (3.3%) | 3 (2.2%) | 103 (5.6%) | <0.001 |
| Episodic hypotension | 42 (8.2%) | 12 (3.6%) | 4 (3.7%) | 4 (2.0%) | 24 (4.9%) | 4 (2.9%) | 96 (5.2%) | 0.0055 |
|  |  |  |  |  |  |  |  |  |
| **Sustained elevated shock index** | 133 (25.9%) | 45 (13.7%) | 19 (17.6%) | 20 (9.8%) | 83 (17.1%) | 18 (13.1%) | 326 (17.8%) | <0.001 |
|  |  |  |  |  |  |  |  |  |
| **Hypoxia with respiratory distress** | 220 (42.9%) | 121 (36.8%) | 47 (43.5%) | 57 (27.9%) | 151 (31.1%) | 50 (36.5%) | 663 (36.2%) | <0.001 |
| Troponin elevation | 355 (69.2%) | 215 (65.3%) | 73 (67.6%) | 145 (71.1%) | 336 (69.3%) | 97 (70.8%) | 1260 (68.8%) | 0.754 |
| **Natriuretic peptide elevation** | 327 (63.7%) | 178 (54.1%) | 68 (63.0%) | 112 (54.9%) | 271 (55.9%) | 60 (43.8%) | 1048 (57.2%) | 0.0015 |
| **Bleeding Risk Assessment, N (%)** |  |  |  |  |  |  |  |  |
| High | 107 (20.9%) | 43 (13.1%) | 20 (18.5%) | 20 (9.8%) | 47 (9.7%) | 10 (7.3%) | 248 (13.5%) | <0.001 |
| Moderate | 238 (46.4%) | 173 (52.6%) | 60 (55.6%) | 116 (56.9%) | 234 (48.2%) | 71 (51.8%) | 915 (49.9%) |  |
| Low | 168 (32.7%) | 113 (34.3%) | 28 (25.9%) | 68 (33.3%) | 204 (42.1%) | 56 (40.9%) | 669 (36.5%) |  |
|  |  |  |  |  |  |  |  |  |
| **Treatment related major bleeding (not ICH)** | 56 (10.9%) | 22 (6.7%) | 8 (7.4%) | 5 (2.5%) | 27 (5.6%) | 9 (6.6%) | 130 (7.1%) | <0.001 |
|  |  |  |  |  |  |  |  |  |
| **Intracranial hemorrhage** | 3 (0.6%) | 2 (0.6%) | 3 (2.8%) | 0 (0%) | 2 (0.4%) | 0 (0%) | 10 (0.5%) | 0.139 |
|  |  |  |  |  |  |  |  |  |
| **Treatment related major bleeding (of any type)** | 59 (11.5%) | 24 (7.3%) | 10 (9.3%) | 5 (2.5%) | 29 (6.0%) | 9 (6.6%) | 139 (7.6%) | 0.002 |
|  |  |  |  |  |  |  |  |  |
| **PE-related death** | 44 (8.6%) | 12 (3.6%) | 10 (9.3%) | 5 (2.5%) | 16 (3.3%) | 5  (3.6%) | 94 (5.1%) | 0.0015 |
|  |  |  |  |  |  |  |  |  |
| **In hospital Cardiac Arrest requiring CPR** | 52 (10.1%) | 16 (4.9%) | 8 (7.4%) | 4 (2.0%) | 18 (3.7%) | 6 (4.4%) | 105 (5.7%) | <0.001 |
|  |  |  |  |  |  |  |  |  |
| **Respiratory failure intervention** | 72 (14.0%) | 26 (7.9%) | 16 (14.8%) | 8 (3.9%) | 28 (5.8%) | 13 (9.5%) | 164 (9.0%) | <0.001 |
|  |  |  |  |  |  |  |  |  |
| **Requiring vasoactive agents** | 80 (15.6%) | 26 (7.9%) | 14 (13.0%) | 11 (5.4%) | 20 (4.1%) | 5 (3.6%) | 158 (8.6%) | <0.001 |
|  |  |  |  |  |  |  |  |  |
| **ICU level of care?** | 321 (62.6%) | 131 (39.8%) | 45 (41.7%) | 79 (38.7%) | 269 (55.5%) | 95 (69.3%) | 957 (52.2%) | <0.001 |
|  |  |  |  |  |  |  |  |  |
| **Initial systolic blood pressure, mmHg,** Mean (SD) | 125 (25.8) | 133 (24.1) | 131 (23.8) | 136 (22.6) | 133 (23.8) | 136 (25.2) | 131 (24.8) | <0.001 |
| **Lowest systolic blood pressure within 3 hours, mmHg** |  |  |  |  |  |  |  |  |
| Mean (SD) | 112 (25.0) | 119 (23.1) | 114 (24.1) | 123 (22.6) | 120 (22.0) | 123 (23.9) | 118 (23.8) | <0.001 |
| **Systolic blood pressure ever less than 90 mmHg?** |  |  |  |  |  |  |  |  |
| No | 418 (81.5%) | 290 (88.1%) | 97 (89.8%) | 193 (94.6%) | 439 (90.5%) | 128 (93.4%) | 1612 (88.0%) | <0.001 |
| Yes (Episodic [< 15 min]) | 46 (9.0%) | 18 (5.5%) | 4 (3.7%) | 6 (2.9%) | 27 (5.6%) | 6 (4.4%) | 114 (6.2%) |  |
| Yes (Sustained [> 15 minutes]) | 49 (9.6%) | 21 (6.4%) | 7 (6.5%) | 5 (2.5%) | 18 (3.7%) | 3 (2.2%) | 105 (5.7%) |  |
| **Mean initial Heart rate (SD), beats per minute** |  |  |  |  |  |  |  |  |
| Mean (SD) | 106 (22.2) | 101 (21.6) | 106 (20.9) | 101 (20.3) | 104 (21.6) | 106 (20.5) | 104 (21.6) | 0.00332 |
| **Highest heart rate within 3 hours, mean (SD), mmHg** | 111 (22.7) | 105 (21.2) | 109 (20.0) | 105 (20.0) | 107 (21.9) | 109 (20.2) | 108 (21.6) | 0.00141 |
| **Shock Index > 1.0?** |  |  |  |  |  |  |  |  |
| No | 301 (58.7%) | 247 (75.1%) | 79.0 (73.1%) | 162 (79.4%) | 350 (72.2%) | 103 (75.2%) | 1283 (70.0%) | <0.001 |
| Yes (episodic) | 75 (14.6%) | 38 (11.6%) | 10 (9.3%) | 24 (11.8%) | 55 (11.3%) | 16 (11.7%) | 225 (12.3%) |  |
| Yes (sustained) | 137 (26.7%) | 44 (13.4%) | 19 (17.6%) | 18 (8.8%) | 78 (16.1%) | 18 (13.1%) | 322 (17.6%) |  |
| **Highest respiratory rate, Mean (SD**), breaths per minute | 25.8 (9.09) | 24.7 (7.26) | 26.3 (6.56) | 24.5 (14.5) | 24.5 (7.05) | 26.4 (9.80) | 25.1 (9.02) | 0.0436 |
| **Initial oxygen saturation, mean SD, %** | 94.0 (7.61) | 94.9 (5.27) | 93.5 (7.46) | 94.4 (5.49) | 94.6 (5.64) | 93.6 (6.51) | 94.4 (6.33) | 0.177 |
| BMI , Mean (SD) | 33.2 (9.60) | 33.2 (10.0) | 33.8 (12.1) | 33.3 (9.11) | 32.6 (8.46) | 33.8 (7.84) | 33.2 (9.41) | 0.693 |
| BSA, Mean (SD) | 2.08 (0.337) | 2.06 (0.331) | 2.04 (0.309) | 2.10 (0.339) | 2.06 (0.310) | 2.19 (0.328) | 2.08 (0.325) | 0.167 |
| **Presence of co-existing systemic infection** | 25 (4.9%) | 12 (3.6%) | 3 (2.8%) | 6 (2.9%) | 12 (2.5%) | 4 (2.9%) | 64 (3.5%) | 0.489 |
|  |  |  |  |  |  |  |  |  |
| **Severe LV dysfunction** | 18 (3.5%) | 7 (2.1%) | 0 (0%) | 8 (3.9%) | 14 (2.9%) | 2 (1.5%) | 51 (2.8%) | 0.243 |
| **Hypovolemia** | 21 (4.1%) | 4 (1.2%) | 4 (3.7%) | 6 (2.9%) | 11 (2.3%) | 3 (2.2%) | 53 (2.9%) | 0.172 |
| **Renal disease** | 78 (15.2%) | 32 (9.7%) | 12 (11.1%) | 23 (11.3%) | 23 (4.7%) | 10 (7.3%) | 180 (9.8%) | <0.001 |
| **COPD** | 96 (18.7%) | 68 (20.7%) | 26 (24.1%) | 36 (17.6%) | 73 (15.1%) | 21 (15.3%) | 325 (17.7%) | 0.154 |
|  |  |  |  |  |  |  |  |  |
| **Known pulmonary hypertension** | 25 (4.9%) | 22 (6.7%) | 4 (3.7%) | 9 (4.4%) | 21 (4.3%) | 7 (5.1%) | 91 (5.0%) | 0.762 |
|  |  |  |  |  |  |  |  |  |
| **Known hospice or end of life** | 16 (3.1%) | 14 (4.3%) | 8 (7.4%) | 4 (2.0%) | 20 (4.1%) | 5 (3.6%) | 70 (3.8%) | 0.233 |
| **Cardiac arrest** | 35 (6.8%) | 5 (1.5%) | 3 (2.8%) | 0 (0%) | 13 (2.7%) | 3 (2.2%) | 60 (3.3%) | <0.001 |
| **Catecholamine support** | 55 (10.7%) | 13 (4.0%) | 7 (6.5%) | 4 (2.0%) | 12 (2.5%) | 1 (0.7%) | 93 (5.1%) | <0.001 |
|  |  |  |  |  |  |  |  |  |
| **High Flow Nasal Cannula oxygen** | 25 (4.9%) | 17 (5.2%) | 11 (10.2%) | 3 (1.5%) | 13 (2.7%) | 6 (4.4%) | 78 (4.3%) | 0.009 |
| **Elevated RV:LV ratio by CT** | 389 (75.8%) | 260 (79.0%) | 87 (80.6%) | 162 (79.4%) | 382 (78.8%) | 115 (83.9%) | 1436 (78.4%) | 0.111 |
| **RV focused echo performed?** |  |  |  |  |  |  |  |  |
| Yes | 486 (94.7%) | 313 (95.1%) | 97.0 (89.8%) | 190 (93.1%) | 471 (97.1%) | 128 (93.4%) | 1741 (95.0%) | 0.0295 |
| **BNP** |  |  |  |  |  |  |  |  |
| Mean (SD). pg/ml | 357 (475) | 279 (366) | 359 (428) | 336 (554) | 275 (420) | 217 (268) | 305 (434) | 0.00325 |
| **Prior DVT or PE** | 137 (26.7%) | 83.0 (25.2%) | 26.0 (24.1%) | 41.0 (20.1%) | 99.0 (20.4%) | 27.0 (19.7%) | 427 (23.3%) | 0.138 |
|  |  |  |  |  |  |  |  |  |
| **Hospitalization within previous 3 weeks** | 98 (19.1%) | 59 (17.9%) | 12 (11.1%) | 22 (10.8%) | 54 (11.1%) | 10 (7.3%) | 265 (14.5%) | <0.001 |
|  |  |  |  |  |  |  |  |  |
| **Current anticoagulant use** | 73 (14.2%) | 31 (9.4%) | 11 (10.2%) | 17 (8.3%) | 22 (4.5%) | 12 (8.8%) | 170 (9.3%) | <0.001 |
|  |  |  |  |  |  |  |  |  |
| **Recent limb immobilization** | 26 (5.1%) | 14 (4.3%) | 5 (4.6%) | 13 (6.4%) | 21 (4.3%) | 6 (4.4%) | 88 (4.8%) | 0.903 |
|  |  |  |  |  |  |  |  |  |
| **Recent trauma within 4-6 weeks** | 14 (2.7%) | 7 (2.1%) | 5 (4.6%) | (2.0%) | 11 (2.3%) | 2 (1.5%) | 46 (2.5%) | 0.687 |
| Recent surgery requiring mechanical ventilation | 59 (11.5%) | 25 (7.6%) | 14 (13.0%) | 19 (9.3%) | 29 (6.0%) | 12 (8.8%) | 164 (9.0%) | 0.0315 |
|  |  |  |  |  |  |  |  |  |
| **Family history of VTE** | 37 (7.2%) | 28 (8.5%) | 10 (9.3%) | 18 (8.8%) | 57 (11.8%) | 9 (6.6%) | 164 (9.0%) | 0.206 |
|  |  |  |  |  |  |  |  |  |
| **Clotting disorder** | 22 (4.3%) | 10 (3.0%) | 2 (1.9%) | 9 (4.4%) | 12 (2.5%) | 2 (1.5%) | 60 (3.3%) | 0.39 |
|  |  |  |  |  |  |  |  |  |
| **Hormone replacement therapy** | 23 (4.5%) | 22 (6.7%) | 3 (2.8%) | 11 (5.4%) | 41 (8.5%) | 10 (7.3%) | 113 (6.2%) | 0.081 |
| **Tobacco smoking** |  |  |  |  |  |  |  |  |
| Current | 83 (16.2%) | 53 (16.1%) | 27 (25.0%) | 30 (14.7%) | 64 (13.2%) | 16 (11.7%) | 287 (15.7%) | 0.132 |
| Ex smoker (smoked > 100 cigarettes in their lifetime but has not smoked in the last 28 days but less than 12 months) | 24 (4.7%) | 19 (5.8%) | 1 (0.9%) | 5 (2.5%) | 25 (5.2%) | 7 (5.1%) | 84 (4.6%) |  |
| Ex smoker for > 12 months | 109 (21.2%) | 76.0 (23.1%) | 25 (23.1%) | 45 (22.1%) | 108 (22.3%) | 24 (17.5%) | 397 (21.7%) |  |
| Never | 296 (57.7%) | 180 (54.7%) | 55 (50.9%) | 124 (60.8%) | 287 (59.2%) | 90 (65.7%) | 1061 (57.9%) |  |
| Missing | 1 (0.2%) | 1 (0.3%) | 0 (0%) | 0 (0%) | 1 (0.2%) | 0 (0%) | 3 (0.2%) |  |
| **Malignancy including leukemia** | 84 (16.4%) | 35 (10.6%) | 12 (11.1%) | 22 (10.8%) | 41 (8.5%) | 10 (7.3%) | 210 (11.5%) | 0.0025 |
| **Dementia** | 24 (4.7%) | 30 (9.1%) | 7 (6.5%) | 17 (8.3%) | 46 (9.5%) | 9 (6.6%) | 133 (7.3%) | 0.053 |
|  |  |  |  |  |  |  |  |  |
| **Acquired Immunodeficiency Syndrome** | 5 (1.0%) | 1 (0.3%) | 0 (0%) | 1 (0.5%) | 0 (0%) | 0 (0%) | 10 (0.5%) | 0.218 |
|  |  |  |  |  |  |  |  |  |
| **Metastatic solid tumor** | 68 (13.3%) | 19 (5.8%) | 11 (10.2%) | 15 (7.4%) | 30 (6.2%) | 6 (4.4%) | 153 (8.4%) | <0.001 |
| **PE severity** |  |  |  |  |  |  |  |  |
| Low-risk | 4 (0.8%) | 1 (0.3%) | 0 (0%) | 1 (0.5%) | 3 (0.6%) | 0 (0%) | 9 (0.5%) | <0.001 |
| Intermediate-low | 218 (42.5%) | 184 (55.9%) | 53 (49.1%) | 136 (66.7%) | 278 (57.3%) | 75 (54.7%) | 977 (53.3%) |  |
| Intermediate-high | 222 (43.3%) | 119 (36.2%) | 46 (42.6%) | 61 (29.9%) | 183 (37.7%) | 56 (40.9%) | 707 (38.6%) |  |
| High-risk | 69 (13.5%) | 25 (7.6%) | 9 (8.3%) | 6 (2.9%) | 21 (4.3%) | 6 (4.4%) | 139 (7.6%) |  |
| **Hospital length of stay, days** Mean (SD) | 6.67 (6.92) | 7.25 (24.2) | 5.58 (5.23) | 4.19 (4.01) | 4.44 (3.75) | 4.69 (5.19) | 5.66 (11.4) | 0.00123 |
|  |  |  |  |  |  |  |  |  |
| **Initial Shock index**, Mean (SD) | 0.885 (0.293) | 0.788 (0.234) | 0.840 (0.252) | 0.775 (0.237) | 0.812 (0.244) | 0.811 (0.227) | 0.826 (0.259) | <0.001 |
|  |  |  |  |  |  |  |  |  |
| **Initial respiratory rate, breaths/minute** Mean (SD) | 21.4 (7.81) | 21.1 (4.66) | 22.2 (9.11) | 20.2 (4.11) | 20.5 (4.65) | 22.4 (5.10) | 21.1 (6.03) | <0.001 |
|  |  |  |  |  |  |  |  |  |
| **Lowest oxygen saturation,** Mean (SD), % | 91.7 (9.97) | 92.8 (5.90) | 91.1 (7.53) | 92.7 (5.81) | 92.5 (8.12) | 91.8 (6.67) | 92.2 (7.92) | 0.145 |
|  |  |  |  |  |  |  |  |  |
| **Total Charlson index,** Mean (SD) | 2.02 (2.57) | 1.41 (2.14) | 1.60 (2.33) | 1.45 (2.07) | 1.20 (1.92) | 1.07 (1.72) | 1.51 (2.21) | <0.001 |
| **RV:LV ratio, by echocardiography** |  |  |  |  |  |  |  |  |
| Mean (SD) | 1.41 (0.475) | 1.38 (0.405) | 1.38 (0.396) | 1.34 (0.452) | 1.38 (0.454) | 1.38 (0.357) | 1.38 (0.443) | 0.592 |
| Mean Time from PE diagnosis to first heparin, minutes (SD), minutes | 155 (358) | 148 (664) | 97.13 (254.4) | 73.5 (106) | 90.9 (147) | 67.0 (125) | 115 (388.4) | 0.0085 |
| Mean Time from Team activation to first heparin, (SD), minutes | 119 (343) | 106 (641) | 42.5 (67.3) | 44.9 (91.8) | 67.8 (128) | 53.8 (123) | 83.5 (339) | 0.025 |
| **Clinical deterioration** | 94 (18.3%) | 36 (10.9%) | 21 (19.4%) | 13 (6.4%) | 36 (7.4%) | 14 (10.2%) | 218 (11.9%) | <0.001 |
| **PE severity and bleeding risk profile** |  |  |  |  |  |  |  |  |
| high-risk PE/high bleed | 18 (3.5%) | 5 (1.5%) | 3 (2.8%) | 1 (0.5%) | 5 (1.0%) | 2 (1.5%) | 34 (1.9%) | <0.001 |
| high-risk PE/moderate bleed | 28 (5.5%) | 12 (3.6%) | 5 (4.6%) | 4 (2.0%) | 8 (1.6%) | 3 (2.2%) | 62 (3.4%) |  |
| high-risk PE/low bleed | 23 (4.5%) | 8 (2.4%) | 1 (0.9%) | 1 (0.5%) | 8(1.6%) | 1 (0.7%) | 43 (2.3%) |  |
| intermediate- high risk PE/high bleed | 48 (9.4%) | 16 (4.9%) | 9 (8.3%) | 10 (4.9%) | 24 (4.9%) | 5 (3.6%) | 113 (6.2%) |  |
| intermediate- high risk PE/moderate bleed | 109 (21.2%) | 63 (19.1%) | 20 (18.5%) | 35 (17.2%) | 87 (17.9%) | 31 (22.6%) | 355 (19.4%) |  |
| intermediate- high risk PE/low bleed | 65 (12.7%) | 40 (12.2%) | 17 (15.7%) | 16 (7.8%) | 72 (14.8%) | 20 (14.6%) | 239 (13.0%) |  |
| intermediate- low risk PE/high bleed | 41 (8.0%) | 22 (6.7%) | 8 (7.4%) | 9 (4.4%) | 18 (3.7%) | 3 (2.2%) | 101 (5.5%) |  |
| intermediate- low risk PE/moderate bleed | 100 (19.5%) | 97 (29.5%) | 35 (32.4%) | 76 (37.3%) | 138 (28.5%) | 37 (27.0%) | 494 (27.0%) |  |
| intermediate- low risk PE/low bleed | 81 (15.8%) | 66 (20.1%) | 10 (9.3%) | 52 (25.5%) | 125 (25.8%) | 35 (25.5%) | 391 (21.3%) |  |
|  |  |  |  |  |  |  |  |  |

* Abbreviations: BMI = body mass index, BNP = brain natriuretic peptide, BSA = body surface area; COPD = Chronic obstructive pulmonary disease, CT = computed tomography; DVT = deep venous thrombosis; ECMO =extracorporeal membrane oxygenation, ICH = intracranial hemorrhage, ICU = intensive care unit, LV = left ventricle, PE = pulmonary embolism; RV = right ventricle; RV:LV = right ventricle to left ventricle diameter ratio, VTE = venous thromboembolism

^†^ Other Hospitals = A composite of a few low enrolling (< 100 patients) sites.
