# Supplementary material for: Comparison of Treatment Approaches and Subsequent Outcomes within a Pulmonary Embolism Response Team Registry
Source: Crit Care Res Pract. 2024 Mar 22;2024:5590805. doi: 10.1155/2024/5590805 (PMC10980543; doi:10.1155/2024/5590805)
Supplement: Supplementary Materials — Table S1: classification criteria for pulmonary embolism severity and bleeding risk assessment. Table S2: advanced PE treatment options based on PE severity and bleeding risk at presentation. Table S3: patient characteristics and outcomes grouped by hospital emergency departments. Table S4: supplemental data on patient characteristics by primary outcome (treatment approach). Table S5: multivariate analyses of treatment approach expressed as binary outcome (advanced PE intervention vs. anticoagulation monotherapy). Table S6: probability of treatment completed on PE severity (intermediate/high-risk) and bleeding risk at presentation expressed as percentages with 95% confidence intervals. Table S7: patient characteristics by secondary outcomes. [file 5590805.f1.zip › Table S4.docx]

| **Table S4:** Supplemental data on patient characteristics by primary outcome (treatment approach) | | | | | | |
| --- | --- | --- | --- | --- | --- | --- |
| **Characteristics** | | **Overall**  **N=1832** | **Anticoagulation only,**  **N = 1440** | **Delayed advanced PE intervention (>12 hours)**  **N=113** | **Immediate advanced PE intervention (within 12 hours)**  **N=279** | **Difference**  **95% CI**  **ANOVA or Chi square** |
| Initially Eligible for advanced Intervention, N (%) | | 855 (46.7%) | 564 (39.2%) | 71 (62.8%) | 220 (78.9%) | <0.001 |
| Not Initially Eligible for advanced Intervention, N (%) | | 977 (53.3%) | 876 (60.8%) | 42 (37.2%) | 59 (21.1%) |  |
| Demographics, N (%) | |  | | | | |
| Mean Age (SD), years | | 62.8 (16.0) | 64.3 (15.9) | 57.3 (14.8) | 57.5 (15.5) | <0.001 |
| Gender: Cis Male, n (%) | | 890 (48.6%) | 690 (47.9%) | 61.0 (54.0%) | 139 (49.8%) | 0.438 |
| Gender: Cis Female, | | 942 (51.4%) | 750 (52.1%) | 52.0 (46.0%) | 140 (50.2%) |  |
| Race, N (%) | |  | | | | |
| American Indian/Alaskan | | 16 (0.9%) | 15 (1.0%) | 1 (0.9%) | 0 (0%) | 0.713 |
| Asian | | 5 (0.3%) | 4 (0.3%) | 0 (0%) | 1 (0.4%) |  |
| Black | | 642 (35.0%) | 492 (34.2%) | 40 (35.4%) | 110 (39.4%) |  |
| Other | | 9 (0.5%) | 9 (0.6%) | 0 (0%) | 0 (0%) |  |
| Pacific Islander | | 1 (0.1%) | 1 (0.1%) | 0 (0%) | 0 (0%) |  |
| Missing | | 34 (1.9%) | 28 (1.9%) | 2 (1.8%) | 4 (1.4%) |  |
| White | | 1125 (61.4%) | 891 (61.9%) | 70 (61.9% | 164 (58.8%) |  |
| Ethnicity, N (%) | |  | | | | |
| Ethnicity, Hispanic, N (%) | | 43 (2.3%) | 35 (2.4%) | 1 (0.9%) | 7 (2.5%) | 0.799 |
| Ethnicity Non-Hispanic | | 1704 (93.0%) | 1336(92.8%) | 106(93.8%) | 262 (93.9%) |  |
| Other | | 85 (4.6%) | 69 (4.8%) | 6 (5.3%) | 10 (3.6%) |  |
| Comorbidities, N (%) | |  | | | | |
| Mean BMI (SD) | | 33.2 (9.41) | 32.8 (9.54) | 34.0 (9.53) | 34.6 (8.52) | 0.00969 |
| COPD | | 325 (17.7%) | 262 (18.2%) | 17 (15.0%) | 46 (16.5%) | 0.635 |
| Severe Renal Disease | | 180 (9.8%) | 151 (10.5%) | 5 (4.4%) | 24 (8.6%) | 0.079 |
| Missing | | 1.0 (0.1%) | 1.0 (0.1%) | 0 | 0 |  |
| Pulmonary Hypertension | | 91 (5.0%) | 74 (5.1%) | 4 (3.5%) | 13 (4.7%) | 0.822 |
| Known Hospice or End of Life | | 70 (3.8%) | 60 (4.2%) | 1 (0.9%) | 9 (3.2%) | 0.181 |
| Risk Factors, N (%) | |  | | | | |
| Recent Hospitalization | | 265 (14.5%) | 205 (14.2%) | 16 (14.2%) | 44 (15.8%) | 0.769 |
| Missing | | 2 (0.1%) | 2 (0.1%) | 0 (0%) | 0 (0%) |  |
| Prior PE/DVT | | 427 (23.3%) | 344 (23.9%) | 26 (23.0%) | 57 (20.4%) | 0.48 |
| Limb Immobilization | | 88 (4.8%) | 60 (4.2%) | 6 (5.3%) | 22 (7.9%) | 0.039 |
|  | Missing | 7 (0.4%) | 5 (0.3%) | 0 (0%) | 2 (0.7%) |  |
| Recent Trauma (past 4-6 weeks) | | 46 (2.5%) | 39 (2.7%) | 1 (0.9%) | 6 (2.2%) | 0.578 |
| Recent Surgery | | 164 (9.0%) | 117 (8.1%) | 7 (6.2%) | 40 (14.3%) | 0.0045 |
| Clotting disorder | | 1760 (96.1%) | 45 (3.1%) | 4 (3.5%) | 11(3.9%) | 0.691 |
|  | Missing | 12 (0.7%) | 8 (0.6%) | 2 (1.8%) | 2 (0.7%) |  |
| Tobacco Use | |  |  |  |  |  |
| Tobacco: Current Smoker | | 287 (15.7%) | 225 (15.6%) | 19.0  (16.8%) | 43.0 (15.4%) | 0.122 |
| Ex-Smoker <12 months | | 84 (4.6%) | 65  (4.5%) | 4  (3.5%) | 15 (5.4%) |  |
| Ex-Smoker >12 months | | 397 (21.7%) | 332 (23.1%) | 22  (19.5%) | 43 (15.4%) |  |
| Never | | 1061 (57.9%) | 815 (56.6%) | 68  (60.2%) | 178 (63.8%) |  |
| Missing | | 3 (0.2%) | 3 (0.2%) | 0 (0%) | 0 (0%) |  |
| Malignancy without Metastasis at time of presentation | | 210 (11.5%) | 183 (12.7%) | 9 (8.0%) | 18 (6.5%) | 0.0035 |
| Known Metastatic disease | | 153 (8.4%) | 137 (9.5%) | 9 (8.0%) | 7 (2.5%) | <0.001 |
| Dementia | | 133 (7.3%) | 119 (8.3%) | 2 (1.8%) | 12 (4.3%) | 0.0025 |
| History of AIDS | | 10 (0.5%) | 9 (0.6%) | 0 (0%) | 1(0.4%) | 1 |
| History of Hormone Replacement Therapy | | 113 (6.2%) | 75 (5.2%) | 14 (12.4%) | 24 (8.6%) | 0.0025 |
| Initial Vital signs at presentation, mean (SD) or frequency (%) | |  | | | | |
| Initial HR (bpm) | | 104 (21.6) | 102 (20.8) | 112 (18.3) | 112 (24.4) | <0.001 |
| Initial RR (breaths per minute) | | 25.1 (9.02) | 24.4 (8.59) | 26.4 (9.16) | 28.6 (10.2) | <0.001 |
| Initial O2 sat (%) | | 94.4 (6.33) | 94.7 (6.02) | 93.3 (5.60) | 93.1 (7.86) | <0.001 |
| Initial SBP (mmHg) | | 131 (24.8) | 134 (23.6) | 128 (26.4) | 119 (26.4) | <0.001 |
| Lowest SBP in 3 hours (mmHg) | | 118 (23.8) | 121 (22.2) | 113 (23.0) | 103 (26.1) | <0.001 |
| Initial Shock Index | | 0.826 (0.259) | 0.787 (0.227) | 0.910 (0.251) | 0.992 (0.337) | <0.001 |
| Hemodynamic collapse and return of spontaneous circulation | | 59 (3.2%) | 8 (0.5%) | 3 (2.6% | 48 (17.2%) | <0.001 |
| Imaging/laboratory N (% | |  |  |  |  |  |
| Elevated Troponin | | 1260 (68.8%) | 934 (64.9%) | 94 (83.2%) | 232 (83.2%) | <0.001 |
| Elevated BNP | | 1048 (57.2%) | 803 (55.8%) | 69 (61.1%) | 176 (63.1%) | 0.054 |
| RV:LV > 1.0 on CT | | 1484 (81.0%) | 1146(79.6%) | 99 (87.6%) | 239 (85.7%) | 0.0125 |
| RV Dilatation on Echo | | 403 (22.0%) | 275 (19.1%) | 31 (27.4%) | 97 (34.8%) | <0.001 |
|  | |  |  |  |  |  |
| PE severity at presentation*  N (%) | |  |  |  |  |  |
| High Risk | | 139 (7.6%) | 53 (3.7%) | 7 (6.2%) | 79 (28.3%) | <0.001 |
| Intermediate-High Risk | | 707 (38.6%) | 512 (35.6%) | 62 (54.9%) | 133 (47.7%) |  |
| Intermediate-Low Risk | | 977 (53.3%) | 868 (60.3%) | 44 (38.9%) | 65 (23.3%) |  |
| Low Risk | | 9 (0.5%) | 7 (0.5%) | 0 (0%) | 2 (0.7%) |  |
| Bleeding risk assessment, N (%) | |  |  |  |  |  |
| High | | 248 (13.5%) | 200 (13.9%) | 21 (18.6%) | 27 (9.7%) | <0.001 |
| Moderate | | 915 (49.9%) | 748 (51.9%) | 42 (37.2%) | 125 (44.8%) |  |
| Low | | 669 (36.5%) | 492 (34.2%) | 50 (44.2%) | 127 (45.5%) |  |
| PE severity/bleeding risk profile | |  |  |  |  |  |
| High PE/high bleed | | 34 (1.9%) | 17 (1.2%) | 4(3.5%) | 13 (4.7%) | < 0.001 |
| High PE/moderate bleed | | 62 (3.4%) | 28 (1.9%) | 1 (0.9%) | 33 (11.8%) |  |
| High PE/low bleed | | 43 (2.3%) | 8 (0.6%) | 2 (1.8%) | 33 (11.8%) |  |
| Intermediate-high PE/high bleed | | 113 (6.2%) | 90 (6.3%) | 12 (10.6%) | 11 (3.9%) |  |
| Intermediate-high PE/moderate bleed | | 355 (19.4%) | 267 (18.5%) | 22 (19.5%) | 66 (23.7%) |  |
| Intermediate-high PE/low bleed | | 239 (13.0%) | 155 (10.8%) | 28 (24.8%) | 56 (20.1%) |  |
| Intermediate-low PE/high bleed | | 101 (5.5%) | 93 (6.5%) | 5 (4.4%) | 3 (1.1%) |  |
| Intermediate-low PE/moderate bleed | | 494 (27.0%) | 450 (31.3%) | 19 (16.8%) | 25 (9.0%) |  |
| Intermediate-low PE/low bleed | | 391 (21.3%) | 332 (23.1%) | 20 (17.7%) | 39 (14.0%) |  |
| Mean hospital length of stay, days (SD) | | 5.35 (10.7) | 5.48 (3.21) | 7.35(15.9) | 5.66(11.4) | 0.0264 |

* We found the following proportions for each criterion used to determine PE severity**:** 81% had RV:LV ratio of 1.0 or greater as determined by CT, 22% had RV dilatation by echocardiography, 3.2% arrived in cardiac arrest, 5.0% required vasopressor support at presentation, 5.6% had sustained hypotension, 5.2% had episodic hypotension, 17.8% had sustained elevated shock index, 36.2% had hypoxia with respiratory distress at rest, 68% had elevated troponin, and 57.2% had elevated brain natriuretic peptide levels.
