# Supplementary material for: Comparison of Treatment Approaches and Subsequent Outcomes within a Pulmonary Embolism Response Team Registry
Source: Crit Care Res Pract. 2024 Mar 22;2024:5590805. doi: 10.1155/2024/5590805 (PMC10980543; doi:10.1155/2024/5590805)
Supplement: Supplementary Materials — Table S1: classification criteria for pulmonary embolism severity and bleeding risk assessment. Table S2: advanced PE treatment options based on PE severity and bleeding risk at presentation. Table S3: patient characteristics and outcomes grouped by hospital emergency departments. Table S4: supplemental data on patient characteristics by primary outcome (treatment approach). Table S5: multivariate analyses of treatment approach expressed as binary outcome (advanced PE intervention vs. anticoagulation monotherapy). Table S6: probability of treatment completed on PE severity (intermediate/high-risk) and bleeding risk at presentation expressed as percentages with 95% confidence intervals. Table S7: patient characteristics by secondary outcomes. [file 5590805.f1.zip › Table S5.docx]

**Table S5:** Multivariable analyses of treatment approach expressed as BINARY OUTCOME (advanced PE intervention vs. anticoagulation monotherapy)

|  | **Advanced PE intervention** | | |
| --- | --- | --- | --- |
| *Predictors* | *Odds Ratios* | *Confidence Interval* | *p-value* |
| (Intercept) | 0.13 | 0.08 – 0.22 | <0.001 |
| Intermediate-high risk PE | 3.13 | 2.40 – 4.09 | <0.001 |
| High-risk PE | 14.17 | 9.28 – 21.64 | <0.001 |
| Moderate bleeding risk assessment | 0.54 | 0.41 – 0.70 | <0.001 |
| High bleeding risk assessment | 0.44 | 0.29 – 0.65 | <0.001 |
| Random Effects | | | |
| σ^2^ | 3.29 | | |
| τ_00_ _hosp_ | 0.42 | | |
| ICC | 0.11 | | |
| Number _clinical site_ | 12 | | |
| Observations | 1767 | | |
| Marginal R^2^ / Conditional R^2^ | 0.158 / 0.253 | | |

ICC = intraclass correlation PE= pulmonary embolism
