# Supplementary material for: Comparison of Treatment Approaches and Subsequent Outcomes within a Pulmonary Embolism Response Team Registry
Source: Crit Care Res Pract. 2024 Mar 22;2024:5590805. doi: 10.1155/2024/5590805 (PMC10980543; doi:10.1155/2024/5590805)
Supplement: Supplementary Materials — Table S1: classification criteria for pulmonary embolism severity and bleeding risk assessment. Table S2: advanced PE treatment options based on PE severity and bleeding risk at presentation. Table S3: patient characteristics and outcomes grouped by hospital emergency departments. Table S4: supplemental data on patient characteristics by primary outcome (treatment approach). Table S5: multivariate analyses of treatment approach expressed as binary outcome (advanced PE intervention vs. anticoagulation monotherapy). Table S6: probability of treatment completed on PE severity (intermediate/high-risk) and bleeding risk at presentation expressed as percentages with 95% confidence intervals. Table S7: patient characteristics by secondary outcomes. [file 5590805.f1.zip › Table S6.docx]

| **Table S6**: Probability of treatment completed on PE severity (intermediate/high-risk) and bleeding risk at presentation expressed as percentages with 95% confidence intervals* | | | |
| --- | --- | --- | --- |
| **PE severity**  **at ED presentation** | **Bleeding Risk Assignment at presentation** | | |
|  | High  bleeding risk | Moderate  bleeding risk | Low  bleeding risk |
| High-risk  PE | ACm: 44.44 (34.34, 54.54)  Advanced (delay): 10.81 (8.91, 12.70)  Advanced (immediate): 44.75 (34.64, 54.86) | ACm: 36.68 (28.34, 45.02)  Advanced (delay): 10.59 (8.68, 12.50)  Advanced (immediate): 52.73 (43.93, 61.53) | ACm: 25.52 (18.10, 32.93)  Advanced (delay): 9.44 (7.46, 11.42)  Advanced (immediate): 65.05 (56.55, 73.55) |
| Intermediate-high  PE | ACm: 81.29 (75.89,86.68)  Advanced (delay): 6.42 (4.74, 8.10)  Advanced (immediate) : 12.30 (8.16, 16.44) | ACm: 75.43 (71.65,79.21)  Advanced (delay): 7.71(6.21,9.22)  Advanced (immediate):16.86 (13.74,19.97) | ACm: 64.43 (59.58, 69.28)  Advanced (delay): 9.52 (7.76, 11.27)  Advanced (immediate) : 26.05 (21.75, 30.36) |
| Intermediate-low risk | ACm: 93.87 (91.27, 96.48)  Advanced(delay): 2.66 (1.63, 3.69)  Advanced (immediate) : 3.47 (1.79, 5.15) | ACm: 91.05 ( 88.98, 93.11)  Advanced (delay): 3.64 ( 2.76, 4.53)  Advanced (immediate) : 5.31 ( 3.87, 6.75) | ACm: 84.75 (8.18, 8.77)  Advanced (delay): 5.53 (4.32, 6.74)  Advanced (immediate): 9.73 ( 7.46, 11.99) |

* Abbreviations: PE = pulmonary embolism, ED = emergency department, ACm = anticoagulation monotherapy, Advanced(delay) = intervention (delayed > 12 hours), Advanced (immediate) = intervention (immediate (less than or equal to 12 hours)
