# Supplementary material for: Comparison of Treatment Approaches and Subsequent Outcomes within a Pulmonary Embolism Response Team Registry
Source: Crit Care Res Pract. 2024 Mar 22;2024:5590805. doi: 10.1155/2024/5590805 (PMC10980543; doi:10.1155/2024/5590805)
Supplement: Supplementary Materials — Table S1: classification criteria for pulmonary embolism severity and bleeding risk assessment. Table S2: advanced PE treatment options based on PE severity and bleeding risk at presentation. Table S3: patient characteristics and outcomes grouped by hospital emergency departments. Table S4: supplemental data on patient characteristics by primary outcome (treatment approach). Table S5: multivariate analyses of treatment approach expressed as binary outcome (advanced PE intervention vs. anticoagulation monotherapy). Table S6: probability of treatment completed on PE severity (intermediate/high-risk) and bleeding risk at presentation expressed as percentages with 95% confidence intervals. Table S7: patient characteristics by secondary outcomes. [file 5590805.f1.zip › Table S7.docx]

**Table S7: Patient characteristics by secondary outcomes**

| Characteristics | No Clinical deterioration (N = 1614) | Clinical deterioration  (N = 218) | Difference,  p-value | No Major bleeding  (N = 1702) | Major Bleeding  (N = 130) | Difference, p-value |
| --- | --- | --- | --- | --- | --- | --- |
| Demographics, N (%) |  | | | | | |
| Mean Age (SD) | 62.9 (16.0) | 61.7 (16.1) | 0.288 | 63.1 (16.0) | 59.1 (16.3) | 0.0066 |
| Male | 793 (49.1%) | 97 (44.5%) | 0.22 | 834 (49.0%) | 56 (43.1%) | 0.203 |
| Female | 821 (50.9%) | 121 (55.5%) |  | 868 (51.0%) | 74 (56.9%) |  |
| Race, N (%) |  | | | | | |
| American Indian/Alaskan | 13 (0.8%) | 3 (1.4%) | 0.157 | 16 (0.9%) | 0 (0%) | 0.421 |
| Asian | 5 (0.3%) | 0 (0%) |  | 5 (0.3% | 0 (0%) |  |
| Black | 551 (34.1%) | 91 (41.7%) |  | 585 (34.4%) | 57 (43.8%) |  |
| Other | 8 (0.5%) | 1 (0.5%) |  | 9 (0.5%) | 0 (0%) |  |
| Pacific Islander | 1 (0.1%) | 0 (0%) |  | 1 (0.1%) | 0 (0%) |  |
| Unknown | 28 (1.7%) | 6 (2.8%) |  | 32 (1.9%) | 2 (1.5%) |  |
| White | 1008 (62.5%) | 117 (53.7%) |  | 1054 (61.9%) | 71(54.6%) |  |
|  |  |  |  |  |  |  |
| Ethnicity Hispanic, N (%) | 36 (2.2%) | 7 (3.2%) | 0.487 | 41 (2.4%) | 2 (1.5%) | 0.103 |
| Cardiac arrest at presentation | 3 (0.2%) | 56 (25.7%) | <0.001 | 34 (2.0%) | 25 (19.2%) | <0.001 |
| Hormone Replacement | 100 (6.2%) | 13 (6.0%) | 1.0 |  |  |  |
| Imaging/laboratory metrics | |  | | | | |
| Elevated  troponin ^†^ | 1077 (66.7%) | 183 (83.9%) | <0.001 | 1159 (68.1%) | 101 (77.7%) | 0.0237 |
| Elevated BNP* | 907 (56.2%) | 141 (64.7%) | 0.0195 | 968 (56.9%) | 80 (61.5%) | 0.313 |
| RV:LV > 1.0 on CT | 1320 (81.8%) | 164 (75.2%) | 0.0269 | 1376 (80.8%) | 108 (83.1%) | 0.642 |
| RV Dilation on Echo | 335 (20.8%) | 68 (31.2%) | <0.001 | 366 (21.5%) | 37 (28.5%) | 0.0781 |

* Abbreviations: BNP = brain natriuretic peptide; CT = computed tomography; LV = left ventricle; RV = Right ventricle; RV:LV = right ventricle to left ventricle diameter ratio

^†^ Our institution used the i-STAT BNP test cartridge (Abbott Point of Care, Abbott Park, IL) measured in pg/mL For troponin we used i-STAT cardiac troponin test cartridge (Abbott Point of Care, Abbott Park, IL) for troponin I and measured in ng/mL or high-sensitivity troponin assays. Less than 0.07 ng/mL was considered a normal value for troponin I whereas normal values for high-sensitivity troponin were less than 12 ng/L for females and less than 20 ng/L for males.
